# Differential pro-tumorigenic effects of *Helicobacter pylori* and *Streptococcus anginosus* on AGS cells: contact-dependent versus metabolite-driven mechanisms

**DOI:** 10.1128/mbio.01589-26

**Published:** 2026-07-31

**Authors:** Luan Luan, Xiuling Song, Yunfei Zeng, Yunyun Xie, Yongxuan Hong, Jiawei Tang, Cong Ma, Bing Gu, Liang Wang

**Affiliations:** 1 Laboratory Medicine, Guangdong Provincial People's Hospital (Guangdong Academy of Medical Sciences), Southern Medical University 70570, Guangzhou, Guangdong Province, China; 2 School of Medicine, South China University of Technology 26467, Guangzhou, Guangdong Province, China; 3 The Marshall Centre for Interventions in Infectious Disease, The University of Western Australia 2720, Perth, Western Australia, Australia; 4 Marshall Research Centre for Medical Microbial Biotechnology, Department of Life Sciences, Faculty of Science, The Hong Kong Polytechnic University (PolyU) 26680, Hong Kong, China; 5 School of Agriculture and Food Sustainability, University of Queensland 206227, Brisbane, Australia; 6 School of Health and Medical Sciences, Edith Cowan University 2498, Perth, Western Australia, Australia; Georgia Institute of Technology, Atlanta, Georgia, USA

**Keywords:** gastric cancer, *Helicobacter pylori*, *Streptococcus anginosus*, AGS cells, transcriptomics, metabolomics

## Abstract

Gastric cancer remains a major global health burden, ranking fifth worldwide in both incidence and mortality. While *Helicobacter pylori* is a well-established Group I carcinogen, increasing evidence suggests that non-*H. pylori* bacteria, including *Streptococcus anginosus*, may also contribute to gastric carcinogenesis. However, their comparative pathogenic roles and interactions remain poorly defined. In this study, public databases showed stage-dependent abundance changes of *H. pylori* and *S. anginosus* but no significant correlation during gastric cancer progression. We further quantified both bacteria in gastric fluid samples collected from 500 individuals using a non-invasive gastric string test and in fecal samples from an independent cohort of 500 individuals by qPCR. In the two cohorts, no significant correlations were observed between the two bacterial pathogens, suggesting distinct colonization and pathogenic patterns. To evaluate functional differences, AGS cell co-culture models were established to explore their pro-tumorigenic effects. *H. pylori* predominantly exerted tumor-promoting effects through bacterial cell-associated mechanisms, whereas *S. anginosus* exerted stronger pro-tumorigenic effects via its metabolites. In particular, transcriptomic analysis revealed that proliferation-associated genes, including *DEK* and *RTF1*, were significantly upregulated by 21.9-fold and 19.2-fold, respectively, in cells treated with *Streptococcus anginosus* metabolite (SAM). Metabolomic profiling of SAM identified increased levels of spermidine and polyamine-related metabolites. Among these, N-acetylcadaverine, N-acetyltyrosine, N-acetyltryptophan, and urocanic acid were experimentally validated to significantly promote AGS cell proliferation. Collectively, these findings demonstrate that *H. pylori* and *S. anginosus* drive gastric tumorigenesis through contact-dependent and metabolite-mediated mechanisms, respectively, highlighting bacterial metabolites as emerging contributors to gastric cancer progression.

**IMPORTANCE:**

The gastric microbiota plays a critical role in gastrointestinal health and disease. However, the ecological interactions between *Helicobacter pylori* and non-*H*. *pylori* bacteria remain poorly understood. Among established bacterial pathogens linked to gastric carcinogenesis, *H. pylori* and *Streptococcus anginosus* are recognized as major contributors. In this study, we systematically evaluated infection patterns and potential associations between these two pathogens using public metagenomic data sets and qPCR analysis of clinical samples (feces and gastric fluid) from multicenter cohorts. We found no significant association between their infection statuses (*P* > 0.05), indicating independent colonization patterns and likely differences in their pathogenic mechanisms within the human host. Complementary *in vitro* and cellular analyses further showed that *H. pylori* primarily acts through direct mucosal colonization and virulence factors, whereas *S. anginosus* influences host responses mainly via its metabolic products. These findings demonstrate that *H. pylori* and *S. anginosus* operate through distinct colonization strategies and pathogenic pathways. They underscore the importance of accounting for mechanistic heterogeneity in gastric microbiome research and provide a conceptual framework for future investigations into microbe-driven pathogenesis of gastric disease.

## INTRODUCTION

Gastric cancer (GC) is a major malignancy of the digestive tract, accounting for approximately 4.9% of global cancer incidence and 6.8% of cancer-related deaths, which makes GC rank the fifth worldwide (1). Currently, *H. pylori* is the best-established etiological agent for gastric cancer, which persistently colonizes the human stomach through flagella and virulence factors such as *VacA* and *CagA*, disrupting epithelial integrity, inducing chronic inflammation, and promoting abnormal proliferation (2–4). China is a high-risk region of *H. pylori* prevalence and a high-burden area of GC, contributing nearly half of the newly diagnosed cases and deaths globally (5). Although *H. pylori* infection has been substantially reduced globally in recent years due to improved hygiene and public awareness, the burden of gastric cancer continues high (6, 7), indicating that additional microbial species may contribute to gastric cancer development (8).

The Correa model systematically describes the classical pathological process of gastric carcinogenesis, mainly encompassing the cascade progression from chronic non-atrophic gastritis, atrophic gastritis, intestinal metaplasia, dysplasia, to gastric cancer. This model proposes that long-term infection with *H. pylori* is a primary initiating factor of gastric cancer. *H. pylori* infection induces chronic inflammatory responses, disrupts the gastric mucosal barrier, causes mucosal injury, promotes genetic alterations in epithelial cells, and thereby accelerates malignant transformation and gastric cancer development. Although the Correa model plays an important role in explaining mechanisms of gastric carcinogenesis, it primarily emphasizes the role of *H. pylori* and does not fully account for contributions from other components of the gastric microbiota (9, 10). In recent years, with the advancement of high-throughput sequencing technologies, increasing evidence has demonstrated that the stomach is not a sterile environment but harbors a complex and dynamic microbial community. Studies have shown that during *H. pylori* infection and gastric mucosal pathological progression, the gastric microbiome undergoes significant compositional changes (11, 12). In particular, non-*H*. *pylori* microorganisms originating from the oral cavity or intestinal tract may become enriched and potentially act synergistically in the process of gastric carcinogenesis. These microorganisms may contribute to carcinogenesis not only by producing metabolites, such as nitrosamine-related compounds and short-chain fatty acids, that promote the formation of carcinogenic substances, but also by modulating local inflammatory responses and host immune regulation (13, 14). Among these bacterial pathogens, *Streptococcus anginosus* is significantly enriched in gastritis, intestinal metaplasia, and gastric cancer (15, 16). Recent studies confirm that *S. anginosus* promotes gastric tumorigenesis by activating pro-inflammatory and pro-tumorigenic signaling pathways, enhancing tumor growth and metastasis, and modulating the tumor immune microenvironment (17, 18). However, the relationship between *H. pylori* and *S. anginosus* infections and their distinct pro-tumorigenic mechanisms remains unclear.

In this study, we first integrated gastric mucosal 16S rRNA gene sequencing data from public databases to characterize the dynamic distribution patterns and potential associations between *H. pylori* and *S. anginosus* across different stages of Correa’s cascade. To further validate these observations in clinical settings, we analyzed *H. pylori* and *S. anginosus* in gastric fluid samples (*n* = 500) collected through a non-invasive string test, as well as in an independent fecal cohort (*n* = 500), thereby evaluating their co-infection characteristics in different biological niches. Furthermore, by combining functional assays with transcriptomic and metabolomic analyses, we systematically investigated the distinct pro-tumorigenic effects mediated by bacterial cells and their secreted metabolites in AGS cells. These findings provide a more comprehensive understanding of bacteria-associated gastric carcinogenesis and suggest that *H. pylori* and *S. anginosus* may contribute to intestinal-type gastric cancer progression through distinct mechanisms.

## RESULTS

### Relative abundance and correlation of *H. pylori* and *S. anginosus* across different stages of gastric cancer via public data set analysis

**Fig 1:**
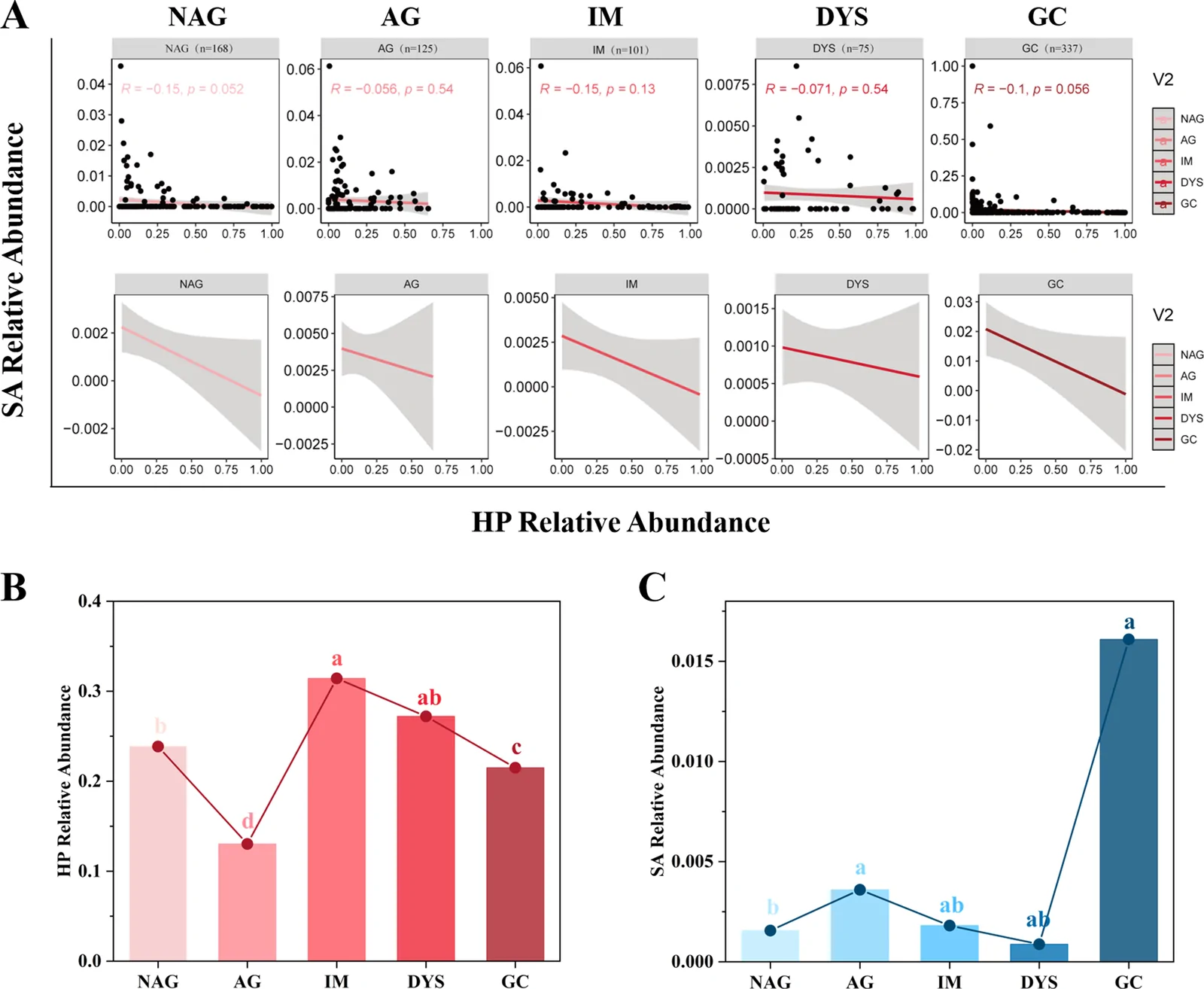
Relative abundance and correlation of *H. pylori* and *S. anginosus* across different stages of Correa’s Cascade from non-atrophic gastritis to gastric cancer. (**A**) Relative abundance correlation between the two bacterial species at different stages of Correa’s cascade. (**B**) Relative abundance of *H. pylori* across gastric cancer stages. (**C**) Relative abundance of *S. anginosus* across gastric cancer stages. Non-atrophic gastritis (NAG), atrophic gastritis (AG), intestinal metaplasia (IM), dysplasia (DYS), and finally gastric cancer (GC). The letters above the bars, a, b, and c, indicate statistically significant differences between groups at *P* < 0.05. Groups sharing the same letter are not significantly different (*P* > 0.05).

To investigate the relative abundance and correlation between *H. pylori* and *S. anginosus*, gastric mucosal 16S rRNA sequencing data (*n* = 806) were retrieved from public database-derived gastric mucosal microbiome data covering different stages of gastric cancer progression via Correa’s cascade, including non-atrophic gastritis (*n* = 168), atrophic gastritis (*n* = 125), intestinal metaplasia (*n* = 101), precancerous lesions (*n* = 75), and gastric cancer (*n* = 337). Detailed methods for collecting and analyzing public database data are provided in Methods S1. Computational analysis revealed that the correlation coefficients between the relative abundances of *H. pylori* and *S. anginosus* were consistently negative across all disease stages, although none reached statistical significance (*P*-value > 0.05), indicating a lack of evidence for coordinated enrichment or depletion along the Correa’s Cascade (Fig. 1A). Notably, the relative abundance of *H. pylori* increased significantly during the intestinal metaplasia stage, followed by a marked reduction in gastric cancer samples (Fig. 1B). In contrast, although *S. anginosus* exhibited a low overall abundance, its relative abundance was significantly elevated in gastric cancer (Fig. 1C). Taken together, these findings indicate that *H. pylori* and *S. anginosus* contribute to gastric carcinogenesis in a stage-specific and temporally distinct manner, rather than through direct synergistic or antagonistic interactions at the mucosal microbial level.

### Correlation of *H. pylori* and *S. anginosus* infection rates in gastric fluid and fecal samples across two independent cohorts

In this study, qPCR was performed on 500 gastric fluid samples collected using a disposable capsule device to evaluate the infection status of *H. pylori* (Hp) and *S. anginosus* (Sa), and logistic regression was used to assess their association. Detailed methods for clinical sample collection and detection analysis are provided in Methods S2. Based on infection status, samples were categorized into four groups: Hp+/Sa+ (112, 22.4%), Hp+/Sa− (72, 14.4%), Hp−/Sa+ (192, 38.4%), and Hp−/Sa− (124, 24.8%). The highest proportion was observed in the Hp−/Sa+ group. The overall positivity rates were 36.8% for Hp and 60.8% for Sa, indicating a higher detection rate of Sa in gastric fluid. Logistic regression analysis showed no significant association between Hp and Sa infection (OR = 1.00, 95% CI: 0.69–1.46, *P* = 1.00), suggesting that Hp status did not influence Sa infection risk in gastric fluid samples. Stratified analyses by sex and age were further performed. In males (*n* = 184), the OR was 0.82 (95% CI: 0.44–1.52, *P* = 0.63). In females (*n* = 316), the OR was 1.12 (95% CI: 0.70–1.79, *P* = 0.72). No significant association was observed in either subgroup. For age stratification, subjects were divided into ≤45 years (*n* = 251) and >45 years (*n* = 249). The OR were 0.79 (95% CI: 0.47–1.31, *P* = 0.43) and 1.34 (95% CI: 0.77–2.33, *P* = 0.36), respectively, with no significant associations observed (Table 1).

**TABLE 1:** Infection rates of *H. pylori* and *S. anginosus* in gastric fluid

Characteristic	Hp+/Sa+	Hp+/Sa−	Hp−/Sa+	Hp−/Sa−	Total	OR	95% CI	*P*-value
Total	112 (22.4%)	72 (14.4%)	192 (38.4%)	124 (24.8%)	500	1	(0.69, 1.46)	1
Sex								
Male	43 (23.4%)	26 (14.1%)	77 (41.8%)	38 (20.7%)	184	0.82	(0.44, 1.52)	0.63
Female	69 (21.8%)	46 (14.6%)	115 (36.4%)	86 (27.2%)	316	1.12	(0.70, 1.79)	0.72
Age								
≤45	51 (20.3%)	44 (17.5%)	93 (37.1%)	63 (25.1%)	251	0.79	(0.47, 1.31)	0.43
>45	61 (24.5%)	28 (11.2%)	99 (39.8%)	61 (24.5%)	249	1.34	(0.77, 2.33)	0.36

In fecal samples (*n* = 500), Hp+/Sa+ accounted for 28 cases (5.6%), Hp+/Sa− for 119 cases (23.8%), Hp−/Sa+ for 48 cases (9.6%), and Hp−/Sa− for 305 cases (61.0%). The overall positivity rates were 29.4% for Hp and 15.2% for Sa. Logistic regression showed no significant association between Hp and Sa infection (OR = 1.44, 95% CI: 0.87–2.38, *P* = 0.16). To further evaluate whether sex and age influence the association between *H. pylori* and *S. anginosus* infection, stratified logistic regression analyses were performed. In gastric fluid samples, within the male subgroup (*n* = 184), 43 cases (23.4%) were Hp+/Sa+, 26 cases (14.1%) were Hp+/Sa−, 77 cases (41.8%) were Hp−/Sa+, and 38 cases (20.7%) were Hp−/Sa−. Logistic regression analysis yielded an OR = 0.82 (95% CI: 0.44–1.52; *P* = 0.63), indicating no statistically significant association. In the female subgroup (*n* =316), 69 cases (21.8%) were Hp+/Sa+, 46 cases (14.6%) were Hp+/Sa−, 115 cases (36.4%) were Hp−/Sa+, and 86 cases (27.2%) were Hp−/Sa−. The corresponding OR was 1.12 (95% CI: 0.70–1.79; *P* = 0.72), which was not statistically significant. These results suggest that no significant association between Hp and Sa infection was observed across sex subgroups. Age-stratified analysis was performed using a cutoff of ≤45 years and >45 years. In the ≤45-year group (*n* = 251), 51 cases (20.3%) were Hp+/Sa+, 44 cases (17.5%) were Hp+/Sa−, 93 cases (37.1%) were Hp−/Sa+, and 63 cases (25.1%) were Hp−/Sa−. Logistic regression analysis showed an OR of 0.79 (95% CI: 0.47–1.31; *P* = 0.43). In the >45-year group (*n* = 249), 61 cases (24.5%) were Hp+/Sa+, 28 cases (11.2%) were Hp+/Sa−, 99 cases (39.8%) were Hp−/Sa+, and 61 cases (24.5%) were Hp−/Sa−, with an OR of 1.34 (95% CI: 0.77–2.33; *P* = 0.36). No statistically significant association was observed in either age group, suggesting that age does not modify the relationship between Hp and Sa infection. In fecal samples, sex-stratified analysis showed no significant association, with an OR of 1.16 (95% CI: 0.52–2.61; *P* = 0.72) in males and 1.71 (95% CI: 0.84–3.49; *P* = 0.13) in females. Similarly, age-stratified analysis revealed no statistically significant association in either subgroup, with an OR of 1.32 (95% CI: 0.64–2.70; *P* = 0.46) in the ≤45-year group and 1.58 (95% CI: 0.71–3.50; *P* = 0.25) in the >45-year group (Table 2). Overall, no significant association between *H. pylori* and *S. anginosus* infection was observed across all stratified analyses. Overall, no significant correlation between Hp and Sa infection was observed in either gastric fluid or fecal samples, suggesting that their colonization patterns may be relatively independent. Differences in detection rates between gastric fluid and fecal samples may reflect distinct microbial niches, sampling sites, and bacterial colonization characteristics within the gastrointestinal tract.

**TABLE 2:** Infection rates of *H. pylori* and *S. anginosus* in fecal samples

Characteristic	Hp+/Sa+	Hp+/Sa−	Hp−/Sa+	Hp−/Sa−	Total	OR	95% CI	*P*-value
Total	28 (5.6%)	119 (23.8%)	48 (9.6%)	305 (61.0%)	500	1.44	(0.87, 2.38)	0.16
Sex								
Male	11 (4.8%)	61 (26.6%)	21 (9.2%)	136 (59.4%)	229	1.16	(0.52, 2.61)	0.72
Female	17 (6.3%)	58 (21.6%)	27 (10.0%)	167 (62.1%)	269	1.71	(0.84, 3.49)	0.13
Age								
≤45	13 (4.0%)	72 (22.2%)	29 (8.9%)	211 (64.9%)	325	1.32	(0.64, 2.70)	0.46
>45	15 (8.6%)	47 (26.9%)	19 (10.9%)	94 (53.7%)	175	1.58	(0.71, 3.50)	0.25

### Differential effects of *H. pylori* and *S. anginosus* on AGS cell functions in co-culture

**Fig 2:**
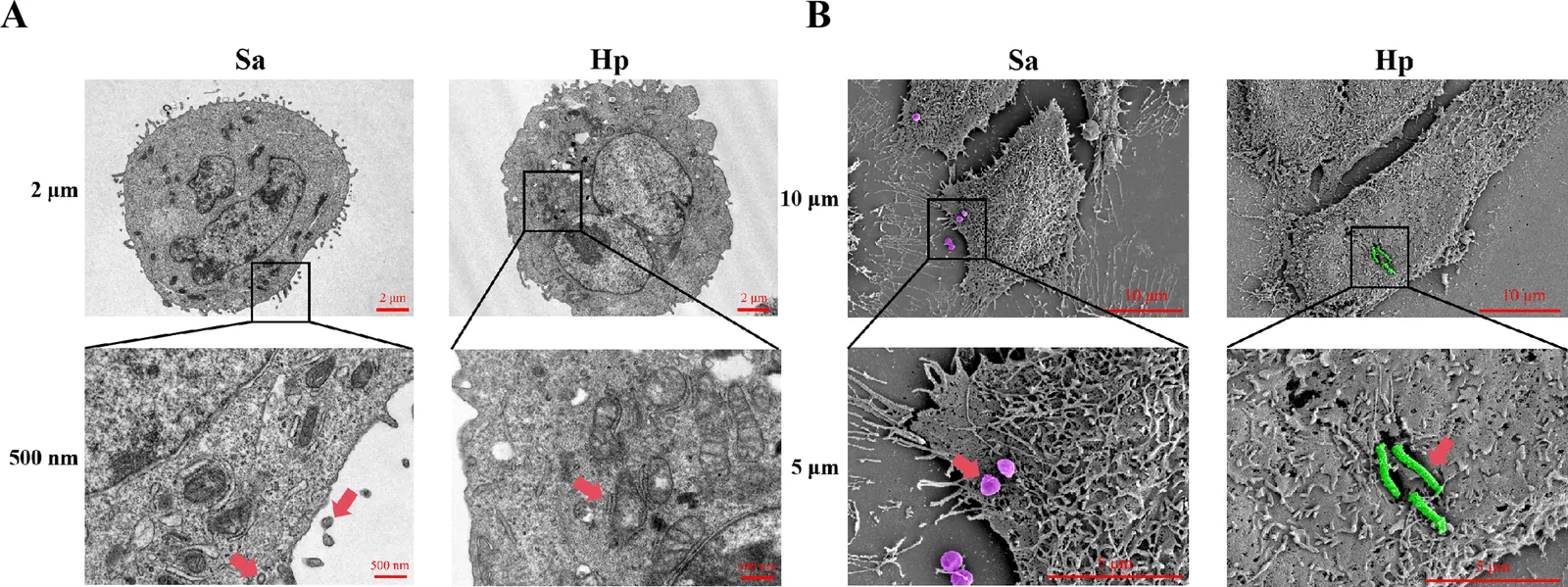
Ultrastructural changes in AGS cells following infection with *H. pylori* and *S. anginosus*. (**A**) TEM. TEM images were acquired at scales of 2 μm and 500 nm. Arrows indicate bacteria. (**B**) SEM. SEM images were acquired at scales of 10 μm and 5 μm. Purple spherical structures represent *S. anginosus* and green curved structures represent *H. pylori*.

To investigate the differential effects of *H. pylori* and *S. anginosus* on gastric epithelial cell function, we cultured and identified the bacteria, established bacterial-cell co-culture models, and further evaluated the effects of bacterial metabolites. The methods for bacterial culture and identification are provided in Methods S3 and S4. *H. pylori* is a Gram-negative curved bacterium, whereas *S. anginosus* is a Gram-positive coccus (Fig. S1). *S. anginosus* exhibited rapid aerobic growth, whereas *H. pylori* displayed slow growth under microaerophilic conditions (Fig. S2). TEM and SEM analyses demonstrated that, after bacterial infection, a large number of bacteria adhered to and aggregated on the surface of AGS cells. In AGS cells, microvilli were reduced or disorganized, and irregular changes in cell margins were observed, suggesting that bacterial adhesion may disrupt membrane structure. In addition, a marked increase in vacuole-like structures in the cytoplasm was observed, indicating that bacterial infection may induce cellular stress responses or structural damage (Fig. 2A and B).

**Fig 3:**
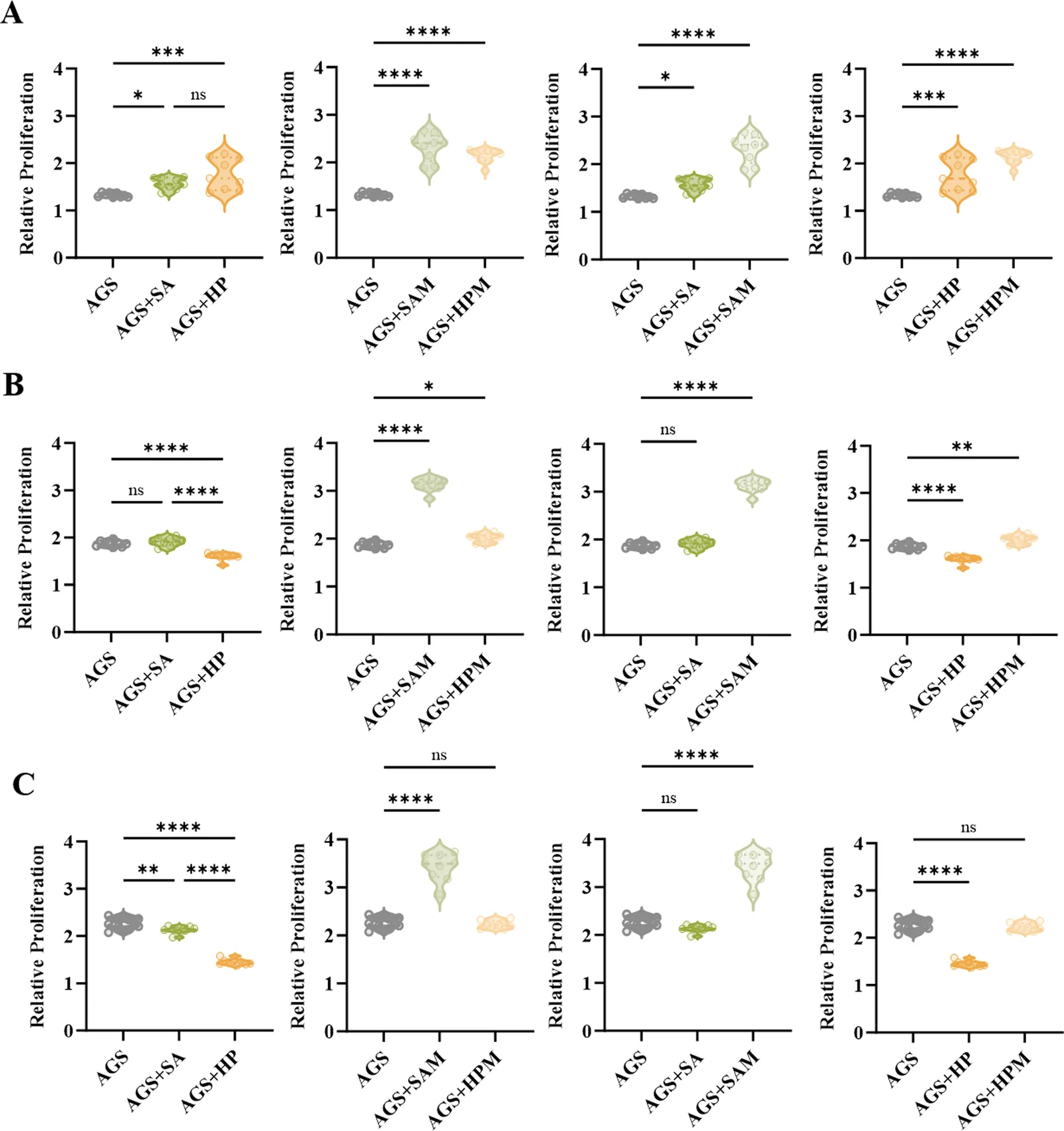
Effects of different treatments on AGS cell proliferation at different time points measured by the CCK-8 assay: (**A**) 24 h, (**B**) 48 h, (**C**) 72 h. *P < 0.05, **P < 0.01, ***P < 0.001, ****P < 0.0001, and ns indicates no statistically significant difference. From left to right in each row, panels represent the bacterial cell groups, the metabolite groups, the *S. anginosus* treatment groups, and the *H. pylori* treatment groups.

**Fig 4:**
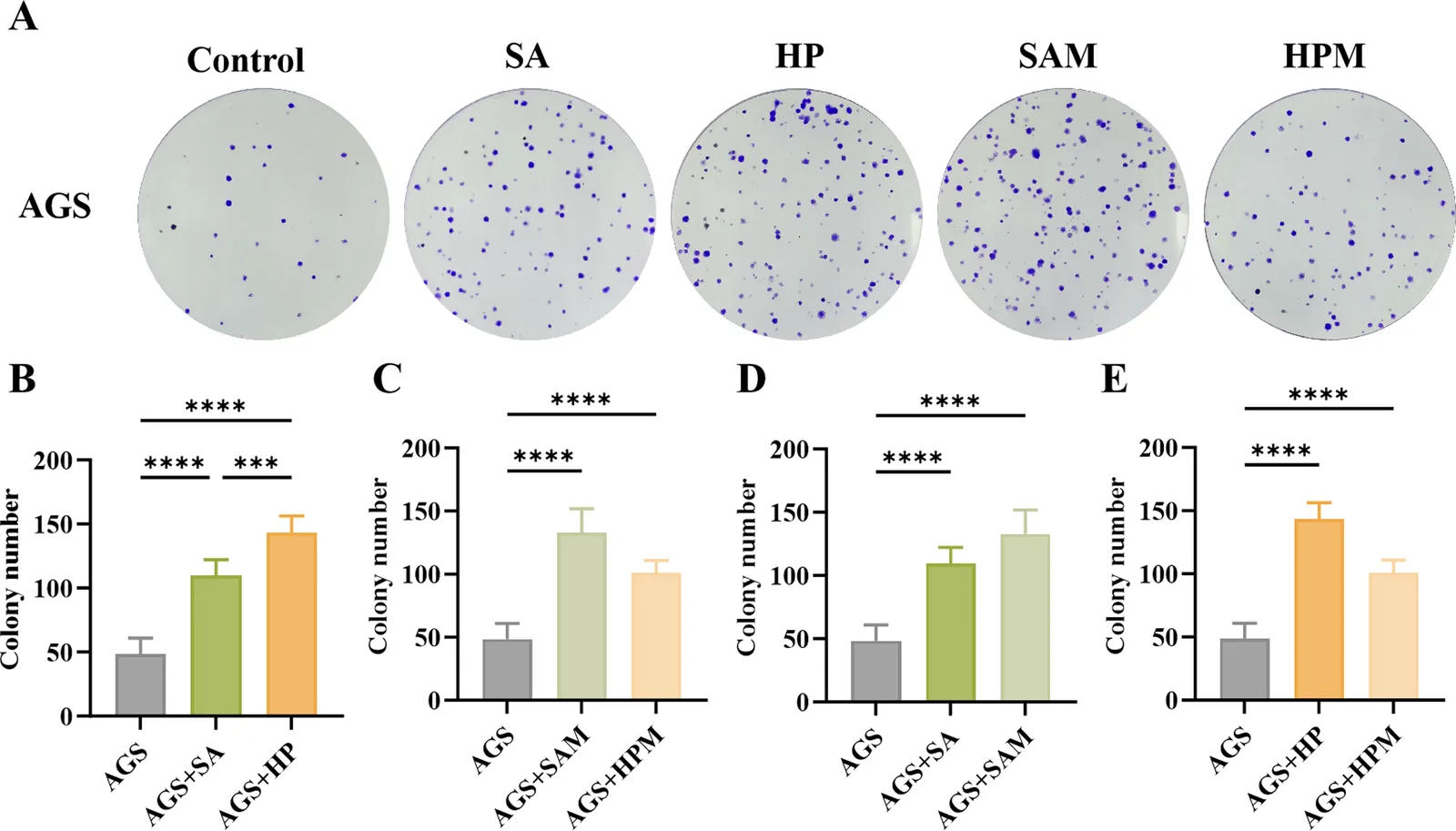
Effects of different treatments on the colony-forming ability of AGS cells. (**A**) Representative images of colony formation assays. (**B–E**) Quantitative analysis of colony formation. (**B**) Bacterial cell groups; (**C**) metabolite groups; (**D**) *S. anginosus* treatment groups; (**E**) *H. pylori* treatment groups. ***P < 0.001 and ****P < 0.0001.

**Fig 5:**
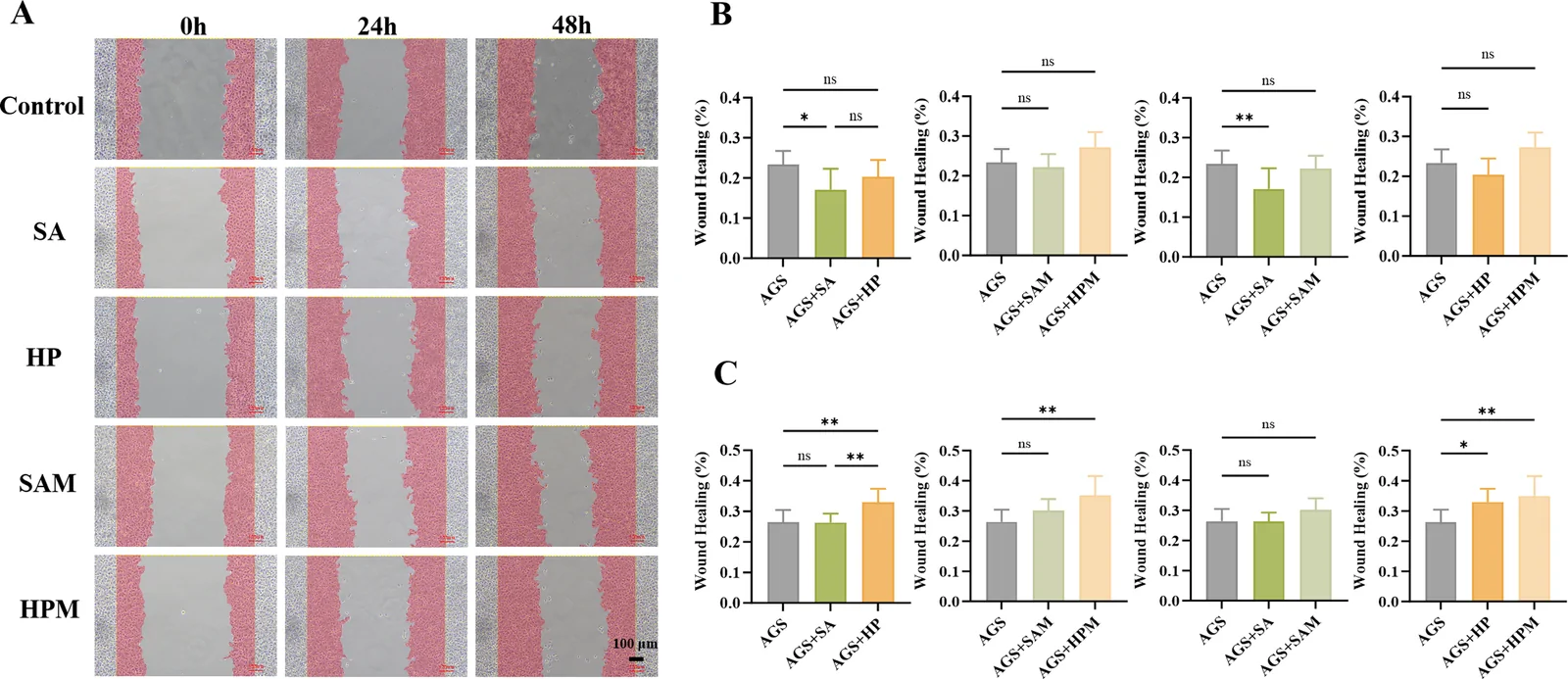
Effects of different treatments on the migration ability of AGS cells. (**A**) Representative images of the wound healing assay at 0 h, 24 h, and 48 h. Scale bar: 100 μm. (**B**) Quantitative analysis at 24 h. (**C**) Quantitative analysis at 48 h. *P < 0.05, **P < 0.01, and ns indicates no statistically significant difference.

**Fig 6:**
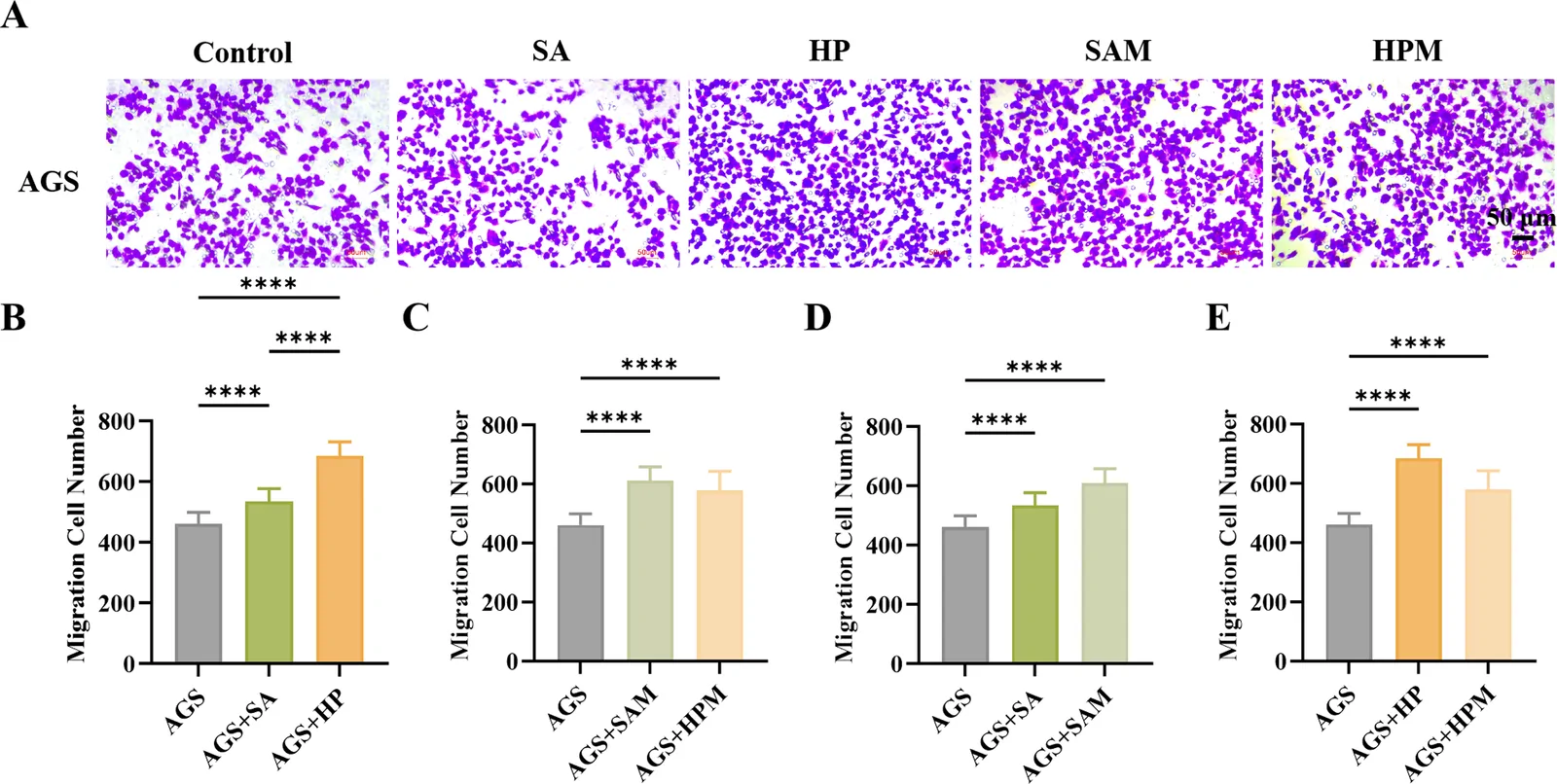
Effects of different treatments on the invasive ability of AGS cells assessed by Transwell assay. (**A**) Representative images of Transwell assay. Scale bar: 50 μm. (**B–E**) Quantitative analysis of invasive ability. (**B**) Bacterial cell groups; (**C**) Metabolite groups; (**D**) *S. anginosus* treatment groups; (**E**) *H. pylori* treatment groups. ****P < 0.0001.

**Fig 7:**
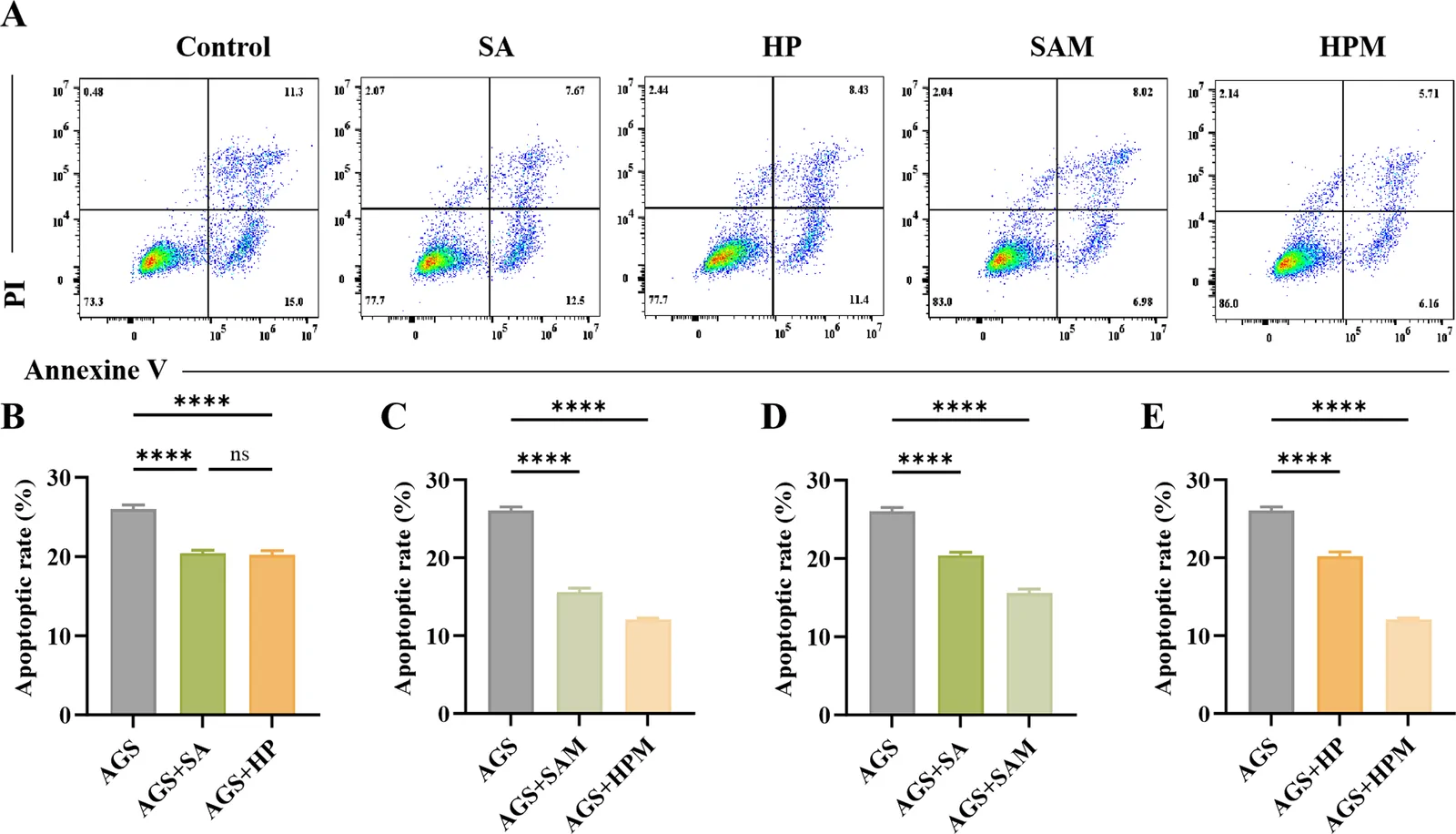
Effects of different bacteria and their metabolites on the apoptosis rate of AGS cells. (**A**) Representative dot plots of apoptosis detected by Annexin V-FITC/PI double staining using flow cytometry. (**B–E**) Quantitative analysis of apoptosis rate of AGS cells. (**B**) Bacterial cell groups; (**C**) metabolite groups; (**D**) *S. anginosus* treatment groups; (**E**) *H. pylori* treatment groups. ****P < 0.0001, and ns indicates no statistically significant difference.

**Fig 8:**
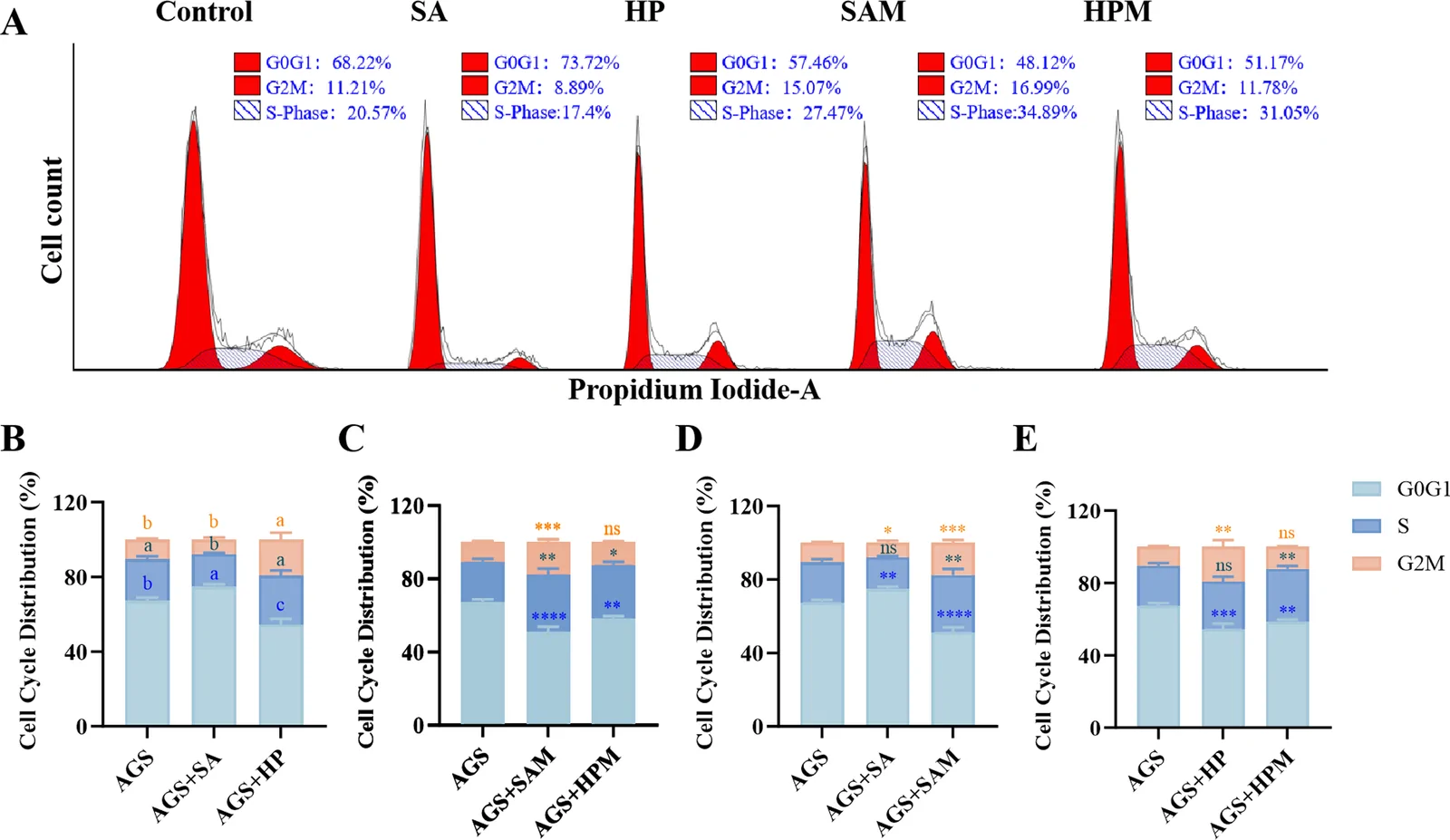
Effects of different bacteria and their metabolites on the cell cycle distribution of AGS cells. (**A**) Representative histograms of cell cycle distribution (G0/G1 phase, S phase, and G2/M phase) analyzed by PI single staining using flow cytometry. (**B–E**) Quantitative analysis of the cell cycle distribution of AGS cells. (**B**) Bacterial cell groups; (**C**) metabolite groups; (**D**) *S. anginosus* treatment groups; (**E**) *H. pylori* treatment groups. The letters above the bars, a, b, and c, indicate statistically significant differences between groups at *P* < 0.05. Groups sharing the same letter are not significantly different (*P* > 0.05). *P < 0.05, **P < 0.01, ***P < 0.001, ****P < 0.0001, and ns indicates no statistically significant difference.

**Fig 9:**
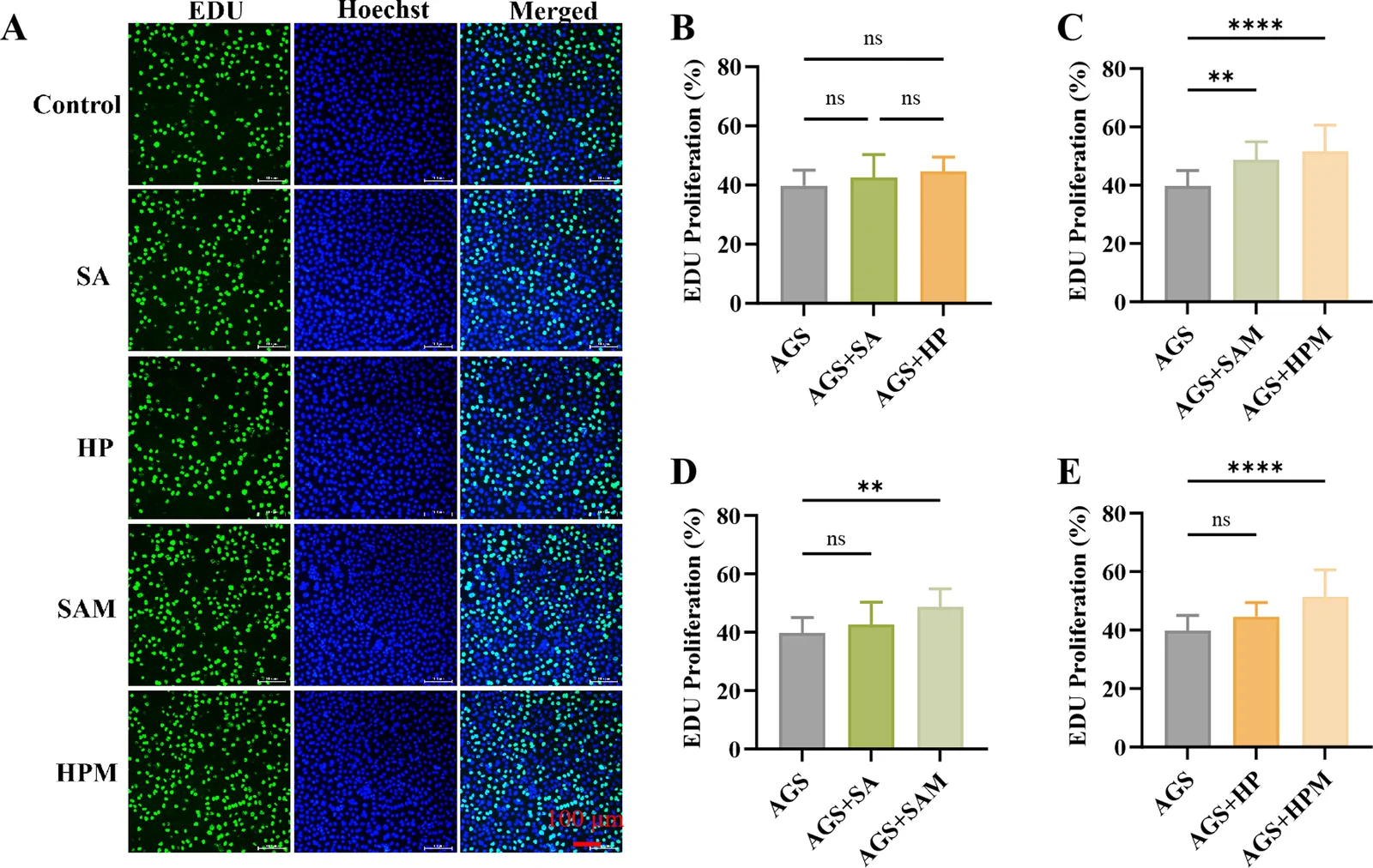
EdU assay for assessing the proportion of S-phase cells in AGS cells under different treatments. (**A**) Representative fluorescence microscopy images of the EdU incorporation assay. Green fluorescence indicates EdU-positive cells (S-phase cells), and blue fluorescence indicates DAPI-stained nuclei (total cells). Scale bar: 100 μm. (**B–E**) Quantitative analysis of the proportion of S-phase cells. (**B**) Bacterial cell groups; (**C**) metabolite groups; (**D**) *S. anginosus* treatment groups; (**E**) *H. pylori* treatment groups. *P < 0.05, **P < 0.01, ***P < 0.001, ****P < 0.0001, and ns indicates no statistically significant difference.

Given the distinct bacterial presence patterns in gastric cancer tissues (19), we next examined whether bacterial cells and their metabolites differentially modulate the malignant phenotypes of AGS cells. CCK-8 results showed that at 24 h, both bacterial cells and their metabolites promoted AGS cell proliferation, with no significant difference between the SA and HP groups (Fig. 3A). At 48 h, SA cells showed no significant effect on AGS cell proliferation, whereas HP cells significantly inhibited cell proliferation. In contrast, both SAM (*Streptococcus anginosus* metabolite) and HPM (*Helicobacter pylori* metabolite) significantly promoted cell proliferation (Fig. 3B). At 72 h, both SA and HP cells significantly inhibited AGS cell proliferation, with a stronger inhibitory effect observed in the HP group. SAM significantly promoted cell proliferation, whereas HPM showed no significant difference compared with the control (Fig. 3C). Overall, the SAM Group promoted AGS cell proliferation, while bacterial cells exhibited time-dependent inhibitory effects. Colony formation assays demonstrated that, compared with the control group, SA, HP, SAM, and HPM groups exhibited an increased number of AGS cell colonies to varying degrees (Fig. 4A). In the bacterial cell groups, the HP group showed a significantly stronger promoting effect than the SA group (Fig. 4B through E). Overall, both bacterial cells and their metabolites promoted colony formation, with HP bacterial cells exerting a more pronounced effect than SA cells. Wound-healing assays showed that *H. pylori* and its metabolites significantly promoted AGS cell migration, whereas *S. anginosus* and its metabolites had no significant effect (Fig. 5). Transwell invasion assays similarly demonstrated that both bacterial suspensions and their metabolites promoted cell invasion, with HP Group showing a significantly stronger effect than SA Group in the bacterial suspension group (Fig. 6). Flow cytometric analysis indicated that *H. pylori* and its metabolites significantly inhibited apoptosis, with a more pronounced effect observed for metabolites (Fig. 7). Cell cycle analysis showed that *H. pylori*, its metabolites, and *S. anginosus* metabolites reduced the proportion of cells in the G0/G1 phase and increased the proportion of S-phase cells, which was further confirmed by EdU incorporation assays (Fig. 8 and Fig. 9). Inflammatory responses were further assessed by qPCR and ELISA. HP markedly increased *IL-8* mRNA expression and protein levels, whereas SA primarily elevated *TNF-α* expression. No significant changes in *IL-6* expression were observed among the SA, HP, SAM, and HPM groups (Fig. S3 to S6).

### Transcriptomic analysis of AGS cells under different treatments

**Fig 10:**
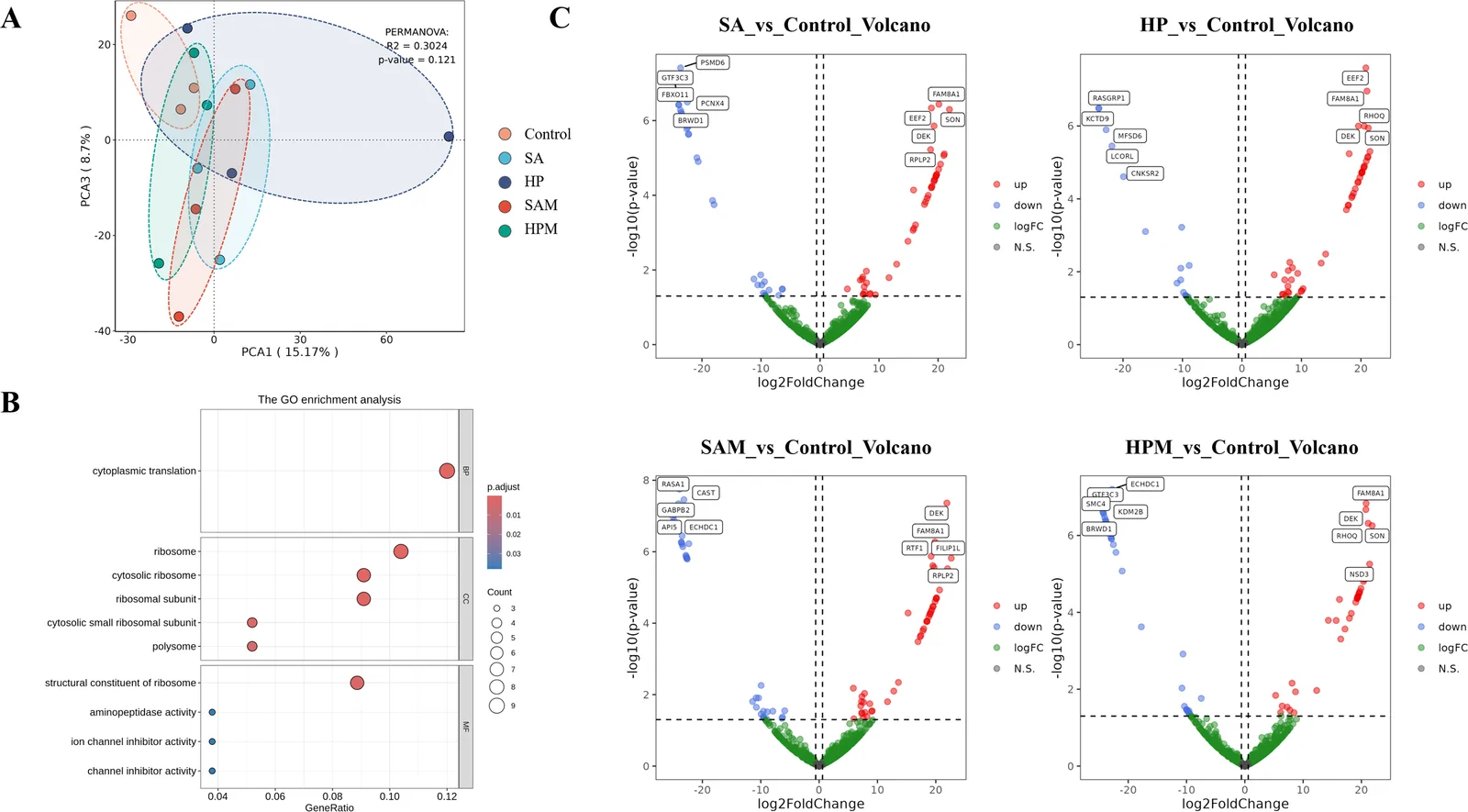
Transcriptomic analysis of AGS cells under different treatments. (**A**) Principal component analysis (PCA) showing the distribution of samples based on the first two principal components (PCA1, 15.17%; PCA2, 15.07%). Each point represents an individual sample. (**B**) GO enrichment analysis of differentially expressed genes. Bubble size indicates the number of enriched genes, and color represents the adjusted *P*-value. (**C**) Volcano plot of differential gene expression between treatment and control groups. The *x*-axis represents log₂(fold change), and the *y*-axis represents −log₁₀(*P*-value). The horizontal dashed line indicates the significance threshold. Red and blue dots denote significantly upregulated and downregulated genes, respectively, while gray dots indicate non-significant genes.

We performed transcriptomic profiling of AGS cells subjected to *H. pylori*, *S. anginosus*, and their metabolites to assess host gene expression changes. Principal component analysis (PCA) showed good intra-group consistency, with the first two principal components explaining 30.24% of the total variance (*R*² = 0.3024). Although a trend toward group separation was observed, it did not reach statistical significance (*P* = 0.121) (Fig. 10A). Compared with untreated controls, *S. anginosus* treatment resulted in 43 upregulated and 30 downregulated genes, while *H. pylori* treatment induced 46 upregulated and 16 downregulated genes. In metabolite-treated groups, *S. anginosus* metabolites altered 82 genes (47 upregulated, 35 downregulated), whereas *H. pylori* metabolites affected 62 genes (35 upregulated, 27 downregulated) (Fig. 10B). Fifteen genes were consistently upregulated across all treatment groups, predominantly associated with transcription and protein synthesis. Among these, *DEK* and *RTF1* were most strongly induced in the SAM group, showing 21.9-fold and 19.2-fold increases, respectively (*P* < 0.05), consistent with the enhanced proliferative phenotypes observed in functional assays. GO enrichment analysis revealed that DEGs were mainly enriched in cytoplasmic translation (biological process), ribosome-related components (cellular component), and ribosomal structural constituents and enzyme regulatory activities (molecular function) (Fig. 10C). KEGG pathway analysis further identified ribosome as the most significantly enriched pathway. These findings indicate that *S. anginosus* metabolites preferentially upregulate ribosome-associated gene expression, which may be associated with the increased proliferation observed in AGS cells.

### Metabolomic analysis of *H. pylori* and *S. anginosus* metabolites

**Fig 11:**
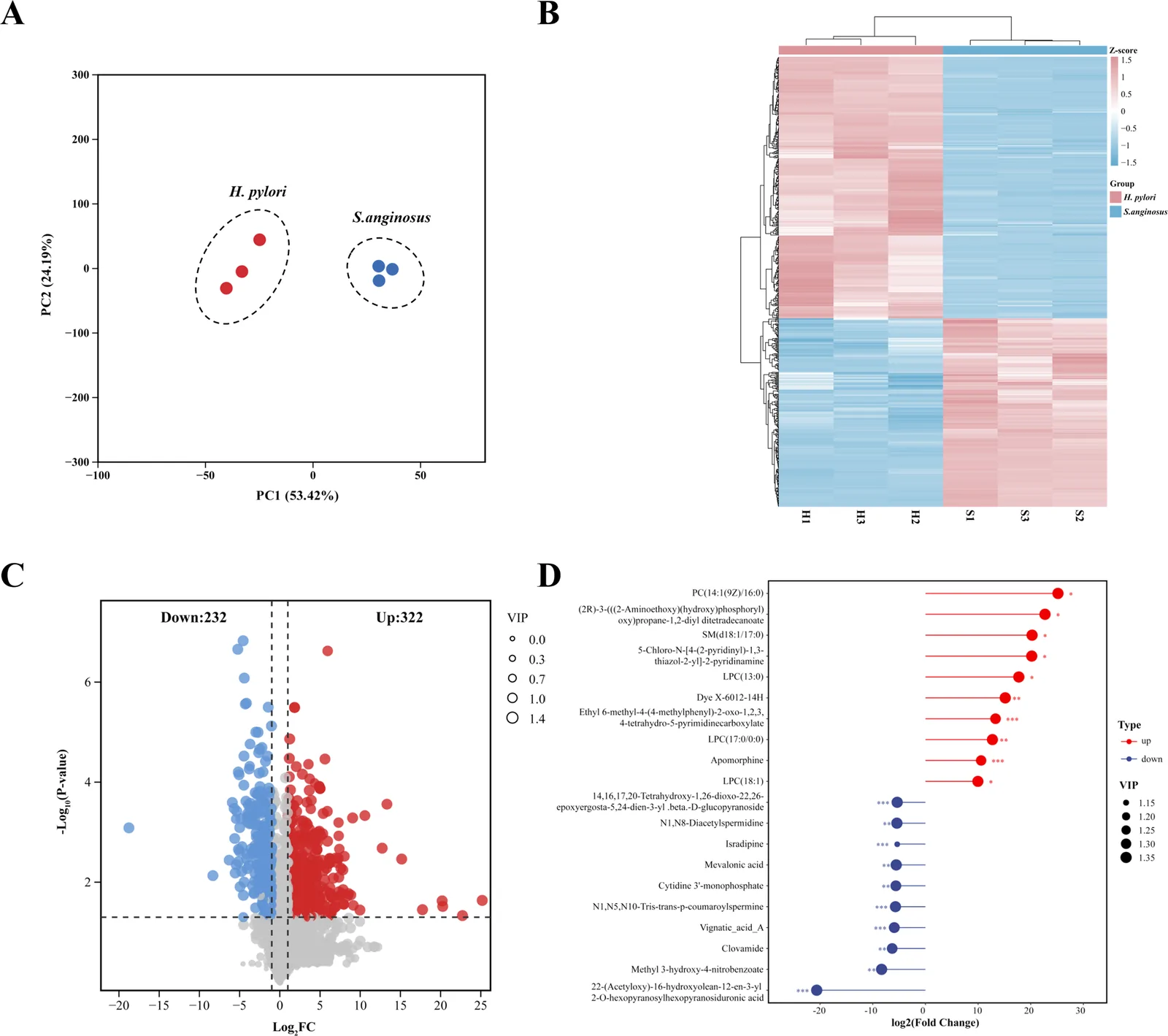
Metabolomic profiling of *H. pylori* and *S. anginosus*. (**A**) PCA score plot showing the separation of metabolite profiles between *H. pylori* (red) and *S. anginosus* (blue) along the first principal component (PC1; 53.42%) and second principal component (PC2; 24.19%). (**B**) Heatmap showing the relative abundance patterns and correlations of metabolites across samples. (**C**) Volcano plot of differential metabolites based on variable importance in projection (VIP > 1) and statistical significance (*P* < 0.05). Red dots indicate metabolites with higher abundance in *H. pylori* relative to *S. anginosus* (Up, *n* = 322), while blue dots indicate metabolites with lower abundance in *H. pylori* compared with *S. anginosus* (Down, *n* = 232). (**D**) Representative examples of the top 10 metabolites enriched in *H. pylori* and *S. anginosus*.

*H. pylori* predominantly exerted tumor-promoting effects through bacterial cell-associated mechanisms, whereas *S. anginosus* exerted stronger pro-tumorigenic effects via its metabolites. Functional assays showed that *S. anginosus* metabolites significantly promoted AGS cell proliferation. The confidence levels of metabolite annotations were evaluated according to the Metabolomics Standards Initiative (MSI) guidelines. Based on evidence from authentic reference standards, MS/MS spectral matching, and database comparison, identified metabolites were classified into different confidence levels. In total, 477 metabolites were confidently identified using authentic reference standards (MSI level 1), supported by consistent accurate mass (MS1), MS/MS fragmentation patterns, and chromatographic retention time (RT). A total of 1,817 metabolites were annotated based on MS/MS spectral matching against public databases, including HMDB, METLIN, and metDNA2, and were classified as MSI level 2 putative identifications. Additionally, 165 metabolites were assigned as MSI level 3 putative candidates based on accurate mass matching and theoretical database annotation, while 93,552 detected features lacked sufficient evidence for reliable annotation and were reported as unknown compounds. To further investigate metabolic differences between *H. pylori* and *S. anginosus*, untargeted metabolomic profiling was performed based on the annotated data set. Principal component analysis (PCA) demonstrated clear separation between the two groups along PC1 (53.42%) and PC2 (21.49%), indicating distinct global metabolic profiles (Fig. 11A). Hierarchical clustering analysis of significantly differential metabolites further revealed clear segregation between the two bacterial groups, suggesting substantial metabolic heterogeneity. Most metabolites exhibited opposite abundance patterns between groups, with metabolites enriched in *H. pylori* showing relatively lower abundance in *S. anginosus*, and vice versa (Fig. 11B). Volcano plot analysis identified 554 significantly differential metabolites (VIP > 1, *P* < 0.05), including 322 metabolites that were significantly higher in *H. pylori* and 232 metabolites that were significantly higher in *S. anginosus* (Fig. 11C). Metabolite classification based on log_2_(fold change) revealed that *H. pylori*-associated metabolites were mainly enriched in phospholipids, amino acid derivatives, and fatty acid-related compounds, whereas *S. anginosus* metabolites were enriched in nucleoside- and amino acid-related derivatives (Fig. 11D). Notably, spermidine and other polyamine-related metabolites were significantly increased in the *S. anginosus* supernatant.

**Fig 12:**
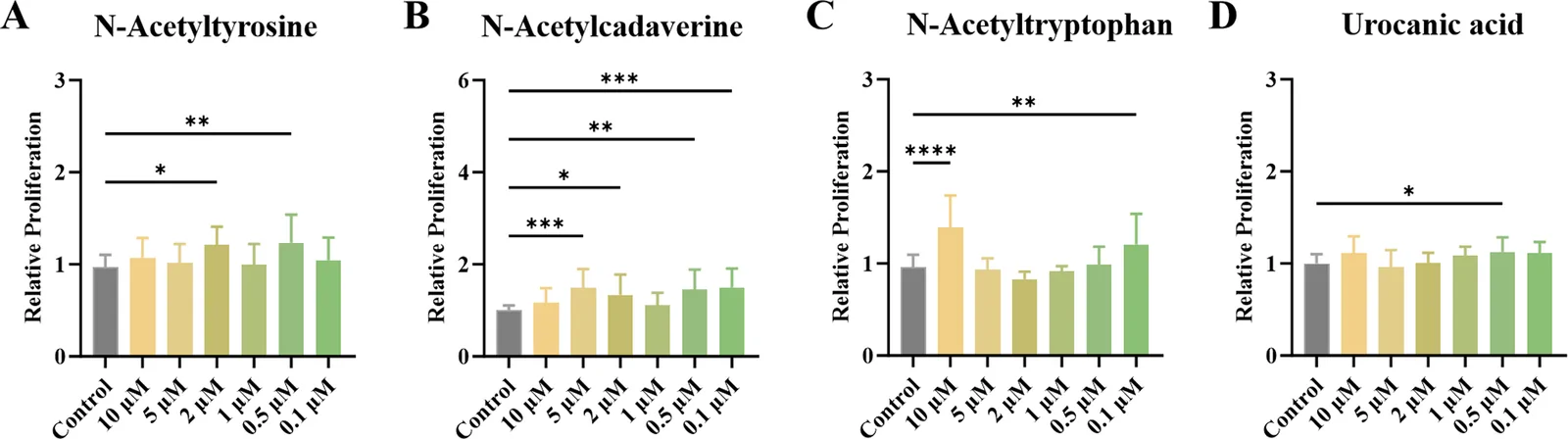
Effects of different concentrations of compounds on AGS cell proliferation. (**A**) Relative proliferation of AGS cells following treatment with N-acetyltyrosine at concentrations of 10, 5, 2, 1, 0.5, and 0.1 μM. (**B**) Relative proliferation of AGS cells following treatment with N-acetylcadaverine at concentrations of 10, 5, 2, 1, 0.5, and 0.1 μM. (**C**) Relative proliferation of AGS cells following treatment with N-acetyltryptophan at concentrations of 10, 5, 2, 1, 0.5, and 0.1 μM. (**D**) Relative proliferation of AGS cells following treatment with Urocanic acid at concentrations of 10, 5, 2, 1, 0.5, and 0.1 μM. *P < 0.05, **P < 0.01, ***P < 0.001, and ****P < 0.0001.

Previous studies have reported that the pro-tumorigenic effects of *S. anginosus* are associated with arginine-derived metabolites involved in amino acid metabolism (18, 20). Consistently, our metabolomic analysis also revealed significant enrichment of arginine derivatives and polyamine-related metabolites in *S. anginosus*. Based on these findings, four amino acid-related metabolites were selected for functional validation (21–23). AGS cells were treated with each metabolite at multiple concentrations (0.1–10 μM). This concentration range was selected based on previously reported *in vitro* studies of amino acid-derived metabolites and polyamines in cancer cell models, where biologically active effects are commonly observed within low- to mid-micromolar ranges, as well as consideration of physiologically relevant levels reported in tumor-associated metabolic environments (23–25). In addition, a preliminary dose-range screening was performed to exclude concentrations that induce non-specific cytotoxicity and to ensure that the selected range covers biologically responsive but non-lethal conditions. AGS cells were then subjected to metabolite treatment, and cell proliferation was subsequently assessed. N-acetyltyrosine significantly promoted AGS cell proliferation at 0.5 μM and 2 μM (Fig. 12A), while N-acetylcadaverine exhibited promotive effects at 0.1, 0.5, 2, and 5 μM (Fig. 12B). N-acetyltryptophan enhanced proliferation at 0.1 μM and 10 μM (Fig. 12C), and urocanic acid promoted proliferation at 0.5 μM (Fig. 12D). Notably, the proliferative effects of these metabolites were not strictly dose-dependent, with growth-promoting activity observed across multiple concentration ranges.

## DISCUSSION

Gastric carcinogenesis is a multistep process shaped by complex host-microbe interactions. Although *H. pylori* is a well-established carcinogen and a key initiator of the Correa’s cascade, its presence alone cannot fully explain the heterogeneity of gastric cancer outcomes. By integrating public database-derived gastric mucosal microbiome data, clinical infection analysis, functional assays, transcriptomics, and metabolomics, this study delineates stage-specific and mechanistically distinct roles of *H. pylori* and *S. anginosus* during intestinal-type gastric cancer progression. Analysis of gastric mucosal 16S rRNA sequencing data across Correa’s cascade showed that *H. pylori* and *S. anginosus* exhibited distinct abundance patterns without significant correlation, suggesting that they do not act synergistically or antagonistically but instead occupy different ecological niches. *H. pylori* abundance peaked at the intestinal metaplasia stage and declined markedly in gastric cancer, whereas *S. anginosus* remained scarce throughout the NAG, AG, IM, and DYS stages of Correa’s Cascade but was significantly enriched in cancer tissues. Collectively, these results are consistent with a two-stage model of gastric carcinogenesis in which *H. pylori* predominantly contributes to tumor initiation, while *S. anginosus* becomes increasingly relevant as tumor development and progression progress (14, 26). This shift likely reflects progressive alterations in the gastric microenvironment. Chronic *H. pylori* infection disrupts acid secretion and mucosal integrity, leading to elevated gastric pH and loss of acid-mediated ecological control (27). These changes permit colonization by microbes from the oral and upper gastrointestinal tracts. The enrichment of *S. anginosus*, a common oral commensal, in gastric cancer tissues is consistent with this ecological transition (12).

Comparison of infection status in gastric fluid and fecal samples revealed substantially higher detection rates of both bacteria in gastric fluid. *H. pylori* is highly adapted to the gastric niche and survives poorly in the distal intestine, explaining its reduced fecal detectability (28, 29). Similarly, *S. anginosus* was more frequently detected in gastric fluid collected using a capsule-based sampling device, which captures the upper gastrointestinal environment *in situ* and minimizes downstream contamination (30, 31). These observations highlight the importance of sampling strategy when investigating gastric microbiota associated with gastric diseases.

Using AGS cells, we systematically compared the functional effects of *H. pylori* and *S. anginosus,* as well as their metabolites. *H. pylori* exerted strong effects through direct bacterium-cell interactions, promoting migration and invasion, inhibiting apoptosis, altering cell cycle distribution, and inducing a pronounced inflammatory response characterized by elevated *IL-8* expression. These findings are consistent with the established role of *H. pylori*-driven inflammation in early gastric tumorigenesis. Previous studies have demonstrated that *H. pylori* infection activates inflammatory signaling pathways and induces pro-inflammatory cytokines such as *IL-8*, thereby promoting a tumor-supportive gastric microenvironment and contributing to gastric carcinogenesis (32, 33). In contrast, *S. anginosus* primarily influenced AGS cell behavior through its secreted metabolites. While bacterial cells had limited effects on migration, *S. anginosus* supernatant significantly promoted cell proliferation, enhanced clonogenic capacity, and increased the proportion of S-phase cells. Inflammatory responses induced by *S. anginosus* differed from those triggered by *H. pylori*, with *TNF-α* rather than IL-8 predominantly upregulated. These results indicate that *S. anginosus* employs a metabolite-dependent mechanism to facilitate tumor-promoting cellular phenotypes.

Transcriptomic analysis revealed that treatment with *S. anginosus* metabolites upregulated genes involved in transcriptional regulation and protein synthesis, including *DEK* and *RTF1*, accompanied by enrichment of ribosome- and polysome-associated pathways. Both genes are well-established regulators of transcriptional elongation and proliferative gene expression programs and have been implicated in gastric cancer progression (34–36). Metabolomic profiling further demonstrated enrichment of arginine- and polyamine-related metabolites, including spermidine, in the *S. anginosus* supernatant. Polyamines play essential roles in maintaining nucleic acid structural stability, regulating cell cycle progression, and promoting cell growth (23). N-acetylcadaverine is a derivative of the polyamine metabolic pathway, and polyamine-related molecules play important roles in maintaining nucleic acid structural stability, regulating cell cycle progression, and promoting cell growth (23). N-acetyltyrosine and N-acetyltryptophan reflect enhanced tyrosine and tryptophan metabolic activity, respectively. Abnormal amino acid metabolism is considered a key component of metabolic reprogramming in tumor cells (37, 38). Urocanic acid, as an intermediate in histidine catabolism, suggests active bacterial histidine metabolism. Its accumulation may indirectly regulate cell survival and proliferation by influencing the local metabolic microenvironment or cellular energy metabolism state (39). Functional validation of selected amino acid-related metabolites confirmed their ability to promote AGS cell proliferation. The non-linear dose responses observed in this study are characteristic of microbiota-derived metabolites that act through context-dependent mechanisms (21, 22). These transcriptomic and metabolomic findings support a model in which *S. anginosus* promotes gastric cancer progression by enhancing host transcriptional and translational capacity through secreted metabolites rather than direct inflammatory stimulation. Collectively, our results support a stage-specific model of gastric carcinogenesis in which *H. pylori* predominantly drives tumor initiation through inflammation and epithelial injury, whereas *S. anginosus* and its metabolites facilitate later-stage progression by sustaining proliferative and biosynthetic programs. This transition coincides with gastric microbial dysbiosis and enrichment of oral-derived taxa in advanced disease.

Although the present study provides important insights, it is not without limitations. First, this study was primarily based on *in vitro* models and a single gastric cancer cell line, further validation in animal models is required to confirm its physiological relevance. Second, in the untargeted metabolomics analysis of bacterial culture supernatants, some annotated compounds were typically reported as diet-related chemicals, plant-derived metabolites, and serum-derived small molecules, which are likely attributable to background components of the culture medium. Last but not least, the selected differential metabolites for downstream analysis were prioritized based on statistical significance and previously reported biological relevance. This highlights the inherent limitations of untargeted metabolomics and the need for cautious interpretation in culture-based systems.

Collectively, these findings support a stage-specific model of microbial contributions to gastric carcinogenesis, in which *H. pylori* primarily drives tumor initiation through direct, contact-dependent mechanisms, whereas *S. anginosus* assumes a progressively more prominent role during tumor progression via metabolite-mediated pathways. This study thereby underscores the critical importance of bacterial metabolites as emerging key contributors to gastric cancer development and progression, revealing a dynamic, temporally regulated interplay between distinct microbial species and host tumorigenesis.

## MATERIALS AND METHODS

### AGS cell culture and co-culture with bacteria

A human adenocarcinoma gastric cell (AGS) line, purchased from Wuhan Pricella Biotechnology Co., Ltd. (China), was authenticated by short tandem repeat (STR) profiling and maintained in complete AGS growth medium (Procell Life Science & Technology Co., Ltd., Wuhan, China), which consisted of Ham’s F-12 medium supplemented with 10% fetal bovine serum (FBS) and 1% penicillin–streptomycin solution. Using the previously described procedure with liquid culture medium, when the culture reached an OD₆₀₀ of 0.5, it was centrifuged at 6,000 rpm for 10 min to collect the supernatant, which was then filtered through a 0.22 μm filter (Biosharp, China) to obtain the clarified supernatant. The bacterial pellet obtained after centrifugation was resuspended in sterile PBS, and the bacterial concentration was adjusted to 10⁸ CFU/mL using a McFarland turbidity standard, yielding a standardized bacterial suspension. AGS cells were cultured following standard subculture procedures until reaching an appropriate cell density. After trypsinization, the cell concentration was adjusted to 10^6^ cells/mL. According to previous studies, the multiplicity of infection (MOI) was set at 100, and the co-culture duration was 24 h (17, 18). For the experiment, 1 mL of cell suspension was seeded into each well of a six-well plate. When the cells reached 70%–80% confluence, the medium containing penicillin and streptomycin was replaced with antibiotic-free medium, and 1 mL of the prepared bacterial suspension was added. The experimental groups included SA, HP, SAM, HPM, and a PBS-treated control group.

### Transmission electron microscopy

Approximately 1 × 10⁶ AGS cells were seeded into six-well plates (Zhenxuan Biotechnology Co., Ltd., Suzhou, China) and co-cultured with bacterial suspension for 24 h. Cells were harvested and fixed overnight with 2.5% glutaraldehyde (Wuhan Servicebio Technology Co., Ltd., Wuhan, China). After three PBS washes for 15 min each, cells were post-fixed with 1% osmium tetroxide (Aladdin Biochemical Technology Co., Ltd., Shanghai, China) for 2 h, dehydrated through a graded ethanol series (30%, 50%, 70%, and 90%), and embedded. Ultrathin sections at 50–70 nm were prepared, stained with 2% uranyl acetate and lead citrate, and imaged using a Hitachi HT7700 transmission electron microscope at 80 kV.

### Scanning electron microscopy

AGS cells were seeded onto coverslips and treated as described above. After graded ethanol dehydration, the samples were dried in a critical-point dryer. Specimens were mounted on sample stubs using conductive carbon tape and sputter-coated with platinum for approximately 120 s. Images were acquired using a Hitachi Regulus 8100 scanning electron microscope.

### Functional assays

#### CCK-8 proliferation assay

Cell proliferation was assessed using a CCK-8 kit (NCM Biotech, China). AGS cells were seeded at a density of 5,000 cells per well in 96-well plates. After treatment with *H. pylori*, *S. anginosus*, and their metabolites for 24, 48, and 72 h, the medium was removed, cells were gently washed with PBS, and AGS complete medium (Wuhan Pricella Biotechnology Co., Ltd., China) containing 10 μL CCK-8 reagent was added to each well. Plates were incubated for 2 h, and absorbance was measured at 450 nm using a microplate reader (Thermo Fisher Scientific, USA). Cell viability was calculated relative to the control group.

#### Colony formation

To evaluate clonogenic ability, 400 treated AGS cells were seeded into six-well plates. The medium was replaced every 3 days. After approximately 2 weeks, colonies were fixed with 4% paraformaldehyde (Servicebio, China) and stained with 0.1% crystal violet (Servicebio, China). Photos of the plates were taken using a smartphone The experiment was repeated six times.

#### Wound healing assay

AGS cells were seeded into six-well plates and grown to full confluence. A linear scratch was created using a 200 μL pipette tip, and the medium was replaced with serum-free medium. Images of the wound area were taken at 0, 24, and 48 h at the same location.

#### Transwell invasion

After 24 h of treatment, AGS cells were harvested, resuspended in serum-free medium, and counted. A total of 3 × 10⁵ cells were seeded into the upper chamber of 24-well Transwell inserts with 8 μm pores coated with Matrigel (Biosharp, China). The lower chamber was filled with 600 μL of medium containing 10% FBS. After 24 h, invaded cells were fixed with 4% paraformaldehyde, stained with 0.1% crystal violet, and imaged using a Leica DMI1 microscope at 20× magnification.

#### EdU assay

Cell proliferation at the S phase was evaluated using an EdU-488 Cell Proliferation Detection Kit (Beyotime Biotechnology, China). EdU working solution was added to the culture medium in 24-well plates, and cells were incubated for 2 h. Cells were fixed with 4% paraformaldehyde, permeabilized with 3% BSA and 0.3% Triton X-100, and subjected to Click reaction solution for 30 min in the dark. Nuclei were counterstained with DAPI, and images were captured using a Leica DMI1 microscope.

#### qPCR for inflammatory cytokines

Total RNA was extracted from treated AGS cells using TRIzol reagent (Takara, Japan) and reverse-transcribed into cDNA using a reverse transcription kit (Takara, Japan). qPCR was performed on a ABI 7500 Real-Time PCR system (Thermo Fisher Scientific, USA). Each reaction was run in triplicate, and relative gene expression was calculated using the 2^⁻ΔΔCt^ method with β*-actin* as the internal control. All primer sequences are provided in Tables S5 and S6.

#### ELISA for inflammatory cytokines

AGS cells were treated as described above, and culture supernatants were collected and centrifuged at 1,000 × *g* for 20 min. Supernatants were diluted 10-fold prior to analysis. ELISA kits (MultiSciences Biotech Co., Ltd., China) were equilibrated to room temperature for 10 min before use. Standard curves were prepared at concentrations of 500, 250, 125, 62.5, 31.2, 15.6, 7.8, and 0 pg/mL. Samples and standards (100 μL per well) were added to the plates and incubated at 37°C for 60 min. After washing, 100 μL biotinylated detection antibody was added and incubated at 37°C for 60 min, followed by washing and incubation with enzyme conjugate for 30 min. After five washes, 90 μL substrate solution was added and incubated at 37°C for 15 min. The reaction was terminated with 50 μL stop solution, and absorbance was immediately measured at 450 nm.

#### Statistical analysis and data visualization

Statistical analyses were performed according to the experimental system grouping:

1. Bacterial groups: control, SA group, and HP group. Differences among groups within the same system were analyzed using one-way analysis of variance (one-way ANOVA), followed by Tukey’s *post hoc* multiple comparison test.
2. Metabolite groups: control, SAM group, and HPM group. Due to differences in culture media between HP- and SA-derived supernatants, each treatment group was independently compared with the PBS control using Student’s *t*-test, without cross-system comparisons.
3. *S. anginosus* treatment groups: control, SA group, and SAM group. Because bacterial suspension and supernatant belong to different experimental systems, statistical comparisons were performed only between each treatment group and the PBS control using *t*-tests, and no direct comparison between bacterial cells and supernatant was conducted.
4. *H. pylori* treatment groups: control, HP group, and HPM group. The statistical approach was the same as in reference 3.

All statistical analyses and data visualization were performed using GraphPad Prism (version 10). The letters above the bars, a, b, and c, indicate statistically significant differences between groups at *P* < 0.05. Groups sharing the same letter are not significantly different (*P* > 0.05). Asterisks indicate statistical significance as follows: *P < 0.05, **P < 0.01, ***P < 0.001, ****P < 0.0001, and ns indicates no statistically significant difference.

### RNA sequencing and transcriptomic analysis

Total RNA was extracted from AGS cells subjected to different treatments using an RNA extraction kit. RNA libraries were constructed using the NEBNext Ultra II RNA Library Prep Kit and sequenced on an Illumina NovaSeq 2500 platform. Differentially expressed genes were identified through rigorous bioinformatic analysis using DESeq2. Pathway enrichment analyses were performed using the Kyoto Encyclopedia of Genes and Genomes (KEGG) and Gene Ontology (GO) databases to elucidate molecular pathways and biological functions affected by co-culture with *H. pylori* and *S. anginosus* and their metabolites.

### Metabolomics analysis

Metabolites from *H. pylori* and *S. anginosus* supernatants were collected as described above and stored at −80°C until analysis. Samples were thawed on ice prior to extraction. Metabolite extraction was performed by mixing 100 μL of sample with 400 μL of pre-chilled extraction solvent (methanol:acetonitrile = 1:1, vol/vol) containing isotopically labeled internal standards. After vortexing, samples were processed using a standardized protein precipitation workflow, and the resulting supernatants were collected for LC–MS analysis. Three biological replicates were included for each group. Chromatographic separation and mass spectrometry analysis were performed using an UHPLC–HRMS system. All detailed instrument settings, chromatographic conditions, and acquisition parameters are provided in Methods S5. Raw data were converted to mzXML format using ProteoWizard software. Feature detection, alignment, and extraction were performed using a standardized data processing pipeline, as described in Methods S5. Metabolite annotation was carried out by matching MS1 accurate mass and MS/MS spectra against the DB 3.0 database. The processed data were subsequently subjected to statistical analysis and visualization for downstream metabolomic interpretation.

### Functional validation of differential metabolites

To investigate the functional effects of differential metabolites on AGS cells, metabolites associated with amino acid metabolism were selected. Stock solutions (100 mM) were prepared in dimethyl sulfoxide (DMSO) and stored at −20°C. Working solutions were freshly prepared by serial dilution in complete culture medium. AGS cells were seeded at 5 × 10³ cells per well in 96-well plates with 100 μL complete medium. To minimize evaporation, 200 μL PBS was added to the outer wells, and plates were incubated overnight at 37°C in a 5% CO₂ incubator. After cell attachment, the medium was replaced with fresh medium containing different concentrations of metabolites (0, 0.1, 0.5, 1, 5, and 10 μM). Each concentration was tested in six technical replicates. After 24 h treatment, cell viability was assessed using a CCK-8 assay. Absorbance was measured at 450 nm using a microplate reader, and cell viability was normalized accordingly. All experiments were performed in triplicate.

## Data Availability

The transcriptomic data generated in this study have been deposited in the NCBI Sequence Read Archive (SRA) under BioProject accession number PRJNA1404416. The metabolomics data have been deposited in the National Genomics Data Center (NGDC, CNCB) under project accession number PRJCA056157.

## SUPPLEMENTAL MATERIAL

Supplemental figures mbio.01589-26-s0001.docx Fig. S1-S6.

Supplemental text mbio.01589-26-s0002.docx Supplemental methods.

Supplemental tables mbio.01589-26-s0003.docx Tables S1-S6.

Table S7 mbio.01589-26-s0004.xlsx Differential metabolites between the supernatants of *H. pylori* and *S. anginosus*.

ASM does not own the copyrights to Supplemental Material that may be linked to, or accessed through, an article. The authors have granted ASM a non-exclusive, world-wide license to publish the Supplemental Material files. Please contact the corresponding author directly for reuse.
