# Supplementary material for: Differential pro-tumorigenic effects of Helicobacter pylori and Streptococcus anginosus on AGS cells: contact-dependent versus metabolite-driven mechanisms
Source: mBio. 2026 Jul 31;17(9):e01589-26. doi: 10.1128/mbio.01589-26 (PMC13556261; doi:10.1128/mbio.01589-26)
Supplement: Supplementary file 1 [file mbio.01589-26-s0001.docx]

**Figure S1** Identification and morphological characteristics of *Helicobacter pylori* and *Streptococcus anginosus*. (A) Colony morphology of Helicobacter pylori on blood agar and the corresponding mass spectrometry identification results. (B) Colony morphology of Streptococcus anginosus on blood agar and the corresponding mass spectrometry identification results. (C) Gram staining of Helicobacter pylori. (D) Gram staining of Streptococcus anginosus. Magnification: ×1000. Scale bar: 50 μm.

**Figure S2** Growth curves and logistic model fitting analysis of *S. anginosus* and *H. pylori*. (A) Growth curve of *S. anginosus* under standard culture conditions. (B) Logistic model fitting of the growth curve of *S. anginosus*. (C) Growth curve of *H. pylori* under microaerophilic conditions. (D) Logistic model fitting of the growth curve of *H. pylori* .

**Figure S3** Relative *IL-6* mRNA expression in AGS cells under different treatments. (A) Bacterial cell groups; (B) Metabolite groups; (C) *S. anginosus* treatment groups; (D) *H. pylori* treatment groups. * *P* < 0.05 and ns indicates no statistically significant difference.

**Figure S4** Relative *IL-8* mRNA expression in AGS cells under different treatments. (A) Bacterial cell groups; (B) Metabolite groups; (C) *S. anginosus* treatment groups; (D) *H. pylori* treatment groups. ** *P* < 0.01, **** *P* < 0.0001, and ns indicates no statistically significant difference.

**Figure S5** Relative *TNF-α* mRNA expression in AGS cells under different treatments. (A) Bacterial cell groups; (B) Metabolite groups; (C) *S. anginosus* treatment groups; (D) *H. pylori* treatment groups. *** *P* < 0.001, **** *P* < 0.0001, and ns indicates no statistically significant difference.

**Figure S6** IL-8 protein levels in cell culture supernatants measured by ELISA. (A) Bacterial cell groups; (B) Metabolite groups; (C) *S. anginosus* treatment groups; (D) *H. pylori* treatment groups. **** *P* < 0.0001, and ns indicates no statistically significant difference.
