# Supplementary material for: Differential pro-tumorigenic effects of Helicobacter pylori and Streptococcus anginosus on AGS cells: contact-dependent versus metabolite-driven mechanisms
Source: mBio. 2026 Jul 31;17(9):e01589-26. doi: 10.1128/mbio.01589-26 (PMC13556261; doi:10.1128/mbio.01589-26)
Supplement: Supplementary file 2 [file mbio.01589-26-s0002.docx]

### 1. Processing and Analysis of 16S rRNA Sequencing Data of Gastric Mucosal Samples from Public Databases

#### 1.1 Public Dataset Collection

This study systematically retrieved publicly available sequencing datasets from the National Center for Biotechnology Information (NCBI) Sequence Read Archive (SRA). Sequencing projects were selected based on predefined inclusion criteria, specifically targeting gastric mucosal samples with complete clinical staging information. 16S rRNA gene amplicon sequencing data were obtained from multiple public SRA projects, including PRJNA413125, PRJNA310127, PRJEB21497, PRJNA239281, PRJNA428883, PRJNA261299, PRJNA678287, PRJEB26931, PRJNA634837, PRJNA932133, PRJEB11763, PRJNA389357, PRJNA1192252, PRJNA532731, PRJNA641258, PRJNA678413, and PRJNA1145484. These datasets include both single-end (SE) and paired-end (PE) sequencing platforms targeting different hypervariable regions of the 16S rRNA gene (e.g., V5-V6, V3-V4, and V4). The primer sequences used in each study are described in the corresponding original publications. The inclusion criteria were as follows: (1) samples derived from human gastric mucosal tissues; (2) availability of clearly defined pathological staging information; and (3) high-quality sequencing data with complete metadata. In total, 806 samples were included in the final analysis, comprising 168 cases of non-atrophic gastritis (NAG), 125 cases of atrophic gastritis (AG), 101 cases of intestinal metaplasia (IM), 75 cases of dysplasia (DYS), and 337 cases of gastric cancer (GC).

## ****1.2 Data Quality Control****

The gastric mucosal 16S rRNA sequencing data used in this study were obtained from publicly available databases. To ensure consistency in data processing and comparability across different studies, raw sequencing data were uniformly downloaded and reanalyzed using a standardized pipeline. Prior to data retrieval, a comprehensive literature review was conducted to confirm that the selected datasets originated from high-quality studies with rigorous experimental design.

**1.3 Data Processing and Feature Construction**

### 1.3.1 Data Import and Primer Removal

For single-end sequencing data, raw reads were imported into QIIME 2 format using *the* QIIME tools import *function*. For paired-end data, the parameter --type 'SampleData [PairedEndSequencesWithQuality]' was specified during import. Primer sequences were removed using the cutadapt plugin. Specifically, the trim-single command was applied to single-end data, while the trim-paired command was used for paired-end data. Primer sequences were specified according to each dataset (e.g., --p-front and --p-adapter parameters). After primer trimming, sequence quality was assessed using qiime demux summarize.

**1.3.2 Quality Control and ASV Inference**

Quality filtering, denoising, and chimera removal were performed using the DADA2 plugin. For single-end data, the denoise-single command was used with parameters including truncation length (--p-trunc-len) set to 250 bp (adjusted according to sequencing length), trimming of the first 10 bp (--p-trim-left), and a maximum expected error threshold (--p-max-ee) of 2.0. For paired-end data, the denoise-paired command was applied with truncation lengths specified for forward and reverse reads (e.g., --p-trunc-len-f and --p-trunc-len-r), followed by merging of paired reads. Multi-threading was enabled to improve computational efficiency. The output included the ASV feature table (table.qza), representative sequences (rep-seqs.qza), and denoising statistics.

**1.3.3 Taxonomic Classification**

Taxonomic assignment of representative sequences was performed using a pre-trained Naïve Bayes classifier based on the Greengenes2 database (October 2022 release; 2022.10.backbone.full-length.nb.qza). Classification was conducted using the feature-classifier classify-sklearn plugin with 20 threads, generating taxonomic annotations from kingdom to species level for each ASV.

**1.4 Data Extraction and Preparation for Analysis**

From the annotated dataset, the relative abundance of Helicobacter pylori and Streptococcus anginosus was extracted for downstream analysis. Samples were categorized into five groups based on disease stage: non-atrophic gastritis (NAG), atrophic gastritis (AG), intestinal metaplasia (IM), dysplasia (DYS), and gastric cancer (GC). These groupings were used to evaluate the dynamic changes in bacterial abundance and their potential associations across disease progression.

**1.5 Correlation Analysis**

Spearman’s rank correlation analysis was performed to evaluate the association between H. pylori and S. anginosus within each disease stage. Correlation coefficients (R) and corresponding P-values were calculated, and statistical significance was defined as *P* < 0.05.

## ****1.6 Differential Abundance Analysis****

Differences in bacterial relative abundance across disease stages were assessed using the Kruskal–Wallis test. When a significant overall difference was detected, Dunn’s post hoc multiple comparisons test was performed for pairwise group comparisons. All statistical tests were two-sided, with *P* < 0.05 considered statistically significant.

**1.7 Statistical Analysis and Visualization**

All statistical analyses were conducted using R software (version 4.4.2). Two-tailed tests were applied throughout, and *P* < 0.05 was considered statistically significant. Data visualization was performed using the ggplot2 package.

#### 2. Clinical Samples Collection and Analysis

#### 2.1 Clinical Samples Collection

#### All participants undergoing screening via gastric fluid or stool samples were required to fast overnight. The exclusion criteria were as follows: (1) use of antibiotics, bismuth agents, or traditional Chinese medicines with antibacterial activity within the past month, or use of proton pump inhibitors or H_2_ receptor antagonists within the past two weeks; (2) receipt of *H. pylori* eradication therapy within the past three months; (3) pregnancy or lactation; (4) severe cardiac, hepatic, renal, or pulmonary dysfunction, or serious neurological or psychiatric disorders; (5) presence of active gastrointestinal bleeding; (6) history of gastric surgery, gastric ulcer, or advanced tumors with a risk of perforation; (7) poor compliance or inability to cooperate with clinical procedures; and (8) participation in other clinical trials or determination by investigators that the subject was unsuitable for inclusion. Gastric fluid samples were collected using a disposable capsule sampling string. One end of the string containing the capsule was held in the mouth, while the other end was fixed to the cheek with adhesive tape. Participants swallowed the capsule with small sips of water. After approximately 1 h, during which the string fully absorbed gastric fluid, trained medical personnel retrieved the string from the stomach, cut the distal 40 cm of the string, and placed it into a sample preservation tube (Tris/Saline/EDTA). Stool samples were self-collected by participants, with approximately a soybean-sized amount placed into a dedicated stool collection tube.

All samples were obtained from a nationwide cohort of healthy participants and were collected following a standardized operating procedure (1). After collection, gastric fluid and stool samples were immediately placed on dry ice and transported to the laboratory. Samples were then aliquoted and stored at –80 °C until further analysis to preserve metabolic stability. For downstream analyses, all samples were processed using a randomized design and analyzed in batches to minimize potential batch effects.

#### 2.2 DNA Extraction and qPCR Detection

#### DNA was extracted using commercial nucleic acid extraction reagents and a nanomagnetic 96-well fully automated nucleic acid extraction system (Norman Biological technology, China). Gastric fluid samples were homogenized by vortexing, and 200 μL of each sample was transferred into a well, followed by the addition of 20 μL proteinase K (Guangzhou DAAN Gene Co., Ltd., China). Samples were lysed using lysis buffer (Shenzhen Hongmei Diagnostic Technology Co., Ltd., China), and nucleic acids were adsorbed onto the surface of nanomagnetic beads (Guangzhou DAAN Gene Co., Ltd., China). The beads carrying nucleic acids were transferred through multiple washing steps using a magnetic rod (Guangzhou DAAN Gene Co., Ltd., China) and finally eluted to separate nucleic acids from the beads, yielding 70-80 μL of purified DNA. Stool samples were first lysed using a stool-specific nucleic acid lysis buffer (Jiangsu Mole Bioscience Co., Ltd., China). After thorough homogenization, DNA extraction was performed following the same procedure as described for gastric fluid samples. Quantitative PCR (qPCR) was performed to detect *H. pylori* and *S. anginosus* using specific primers and probes. Detection of *H. pylori* was carried out using a commercial kit, and the primer and probe sequences are proprietary. The primer and probe sequences for *S. anginosus* are provided in Table S1. Amplification was carried out under predefined cycling conditions according to the manufacturer’s instructions, and amplification time and reaction parameters were provided in Tables S2-S4.

#### 2.3 Correlation Analysis between *Helicobacter pylori* and *Streptococcus anginosus*

Samples were classified as positive or negative based on Ct values, with a Ct value ≤ the predefined threshold defined as positive, and a Ct value above the threshold or no amplification signal defined as negative. According to the results, samples were categorized into Hp-positive and Hp-negative groups, as well as Sa-positive and Sa-negative groups, and all detection results were treated as binary variables for subsequent statistical analysis. Pearson’s chi-square test (χ² test) was used to compare differences in positivity rates between the two bacteria, and the odds ratio (OR) with 95% confidence interval (95% CI) was calculated to evaluate the association between *H. pylori* and *S. anginosus* infection, with *P* < 0.05 considered statistically significant. To further assess the influence of demographic factors, stratified analyses were performed by sex (male and female) and age (≤ 45 years and > 45 years), with separate 2×2 contingency tables constructed in each stratum to calculate Ors, 95% Cis, and *P* values. In addition, multivariable binary logistic regression was performed, with Sa infection status as the dependent variable and Hp infection status as the independent variable, while adjusting for sex and age as covariates, and adjusted Ors and 95% Cis were calculated. All statistical analyses were performed using SPSS software (version 21); categorical variables were expressed as counts (n) and percentages (%), group comparisons were conducted using the chi-square test, and all tests were two-sided with *P* < 0.05 considered statistically significant.

#### 3. Bacterial Culture and Growth Curves Construction

#### *H. pylori* strain ATCC43504 was provided by the Medical Microbiology Laboratory of Guangdong Provincial People’s Hospital, while *S. anginosus* was a clinically isolated strain. *H. pylori* is a microaerophilic bacterium and was cultured in a sealed jar filled with a mixed gas environment consisting of 6% O₂, 7.2% CO₂, 7.2% H₂, and 79.7% N₂ for 3–5 days. *S. anginosus* is an aerobic bacterium and was cultured in a 5% CO₂ incubator for 24 h. For *S. anginosus* growth curve analysis, single colonies were inoculated into 20 mL of BHI broth and incubated overnight at 37 °C with shaking at 220 rpm. The culture was adjusted to an OD₆₀₀ of 0.5 using a microplate reader. Subsequently, 200 μL of the seed culture was transferred into 20 mL of fresh BHI broth and incubated at 37 °C with shaking at 120 rpm. OD₆₀₀ values were measured at 0, 2, 4, 8, 12, 16, 20, and 24 h. Each experiment was performed in triplicate. For *H. pylori*, a liquid medium was prepared by mixing BHI broth and fetal bovine serum at a 9:1 ratio. Several single colonies were inoculated into the medium and cultured under microaerophilic conditions at 37 °C with shaking at 120 rpm for 24–48 h. The culture was adjusted to an OD₆₀₀ = 0.08. Then, 200 μL of the seed culture was added to 20 mL of fresh medium in a centrifuge tube and incubated in a microaerophilic jar at 37 °C with shaking at 120 rpm for up to 144 h. OD₆₀₀ values were recorded every 12 h. All experiments were repeated three times. Growth curve fitting was performed using a logistic growth model. Curve fitting was conducted using Origin software (OriginLab, USA), and the goodness of fit was evaluated using the coefficient of determination (R²). The fitted logistic curves were used to characterize and compare the bacterial growth dynamics under different conditions.

**4. Mass Spectrometry Identification and Gram Staining of *Helicobacter pylori* and *Streptococcus anginosus***

Single colonies were gently picked using a sterile inoculating loop and applied onto the target plate of the mass spectrometer. Care was taken to evenly spread the sample within the designated area to avoid contaminating neighboring spots. After air-drying for a few seconds, 1 µL of matrix solution (α-cyano-4-hydroxycinnamic acid, CHCA) was added onto each sample and allowed to dry. The quality control strain *Escherichia coli* ATCC 8739 was applied to the central QC position and treated identically to the test samples. Mass spectrometry was performed in RUO mode with a laser frequency of 20-200 Hz and 100 laser shots per spectrum, covering an m/z range of 2,000-20,000 Da. Acquired spectra were matched against the reference database for bacterial identification. The resulting data were exported in txt format and visualized using Origin software (OriginLab, USA).

A few drops of sterile saline solution or broth were placed at the center of a clean glass slide. A single bacterial colony was picked using an inoculation loop and thoroughly mixed with the solution to form a smear, which was then dried in a 65 °C oven and labeled. The smear was stained with crystal violet for 1 min and gently rinsed with running water to remove excess stain, followed by treatment with iodine solution for 1 min and rinsing until no further color change was observed. Decolorization was performed using 95% ethanol for 30 s, followed by rinsing with water until the color disappeared. The slide was then counterstained with safranin for 30 s and gently rinsed to remove excess stain. After drying, the slide was examined under a light microscope using an oil immersion objective (×1000) with immersion oil. Bacteria retaining a purple color were identified as Gram-positive, whereas those appearing red were identified as Gram-negative.

### 5. Metabolomic Analysis of Bacterial Supernatants

**5.1 Metabolite Extraction**

**Metabolites from *H. pylori* (Hp) and *S. anginosus* (Sa) supernatants were collected as described previously and stored at −80 °C until further analysis. Samples were thawed slowly on ice prior to processing and subjected to metabolite extraction using a Starlid™ automated workstation. Each group contained three biological replicates. For each sample, 100 μL of supernatant was mixed with 400 μL of pre-chilled extraction solvent (methanol:acetonitrile = 1:1, v/v) containing a mixture of stable isotope-labeled internal standards. The internal standard mixture consisted of 4-Aminobutyric-2,2,3,3,4,4-d6 acid (2.0 μM), Betaine-(trimethyl-d9) hydrochloride (0.4 μM), Nicotinamide-2,4,5,6-d4 (0.6 μM), Succinic-2,2,3,3-d4 acid (3.0 μM), L-Leucine-d3 (methyl-d3) (8.0 μM), and N-Benzoyl-d5-glycine (0.6 μM). The mixture was transferred to a 96-well protein precipitation plate, vortexed at 750 rpm for 5 min, incubated for 5 min, and then filtered. The resulting filtrate was collected for subsequent LC–HRMS/MS analysis. The isotopically labeled internal standards were included to monitor technical variation across sample preparation, metabolite extraction, and instrumental analysis. Their use enables improved quality control, correction of analytical variability, and enhanced reliability of metabolite detection in untargeted metabolomics workflows. Similar isotope-assisted strategies have been widely applied to improve data robustness and support signal correction based on isotope pattern recognition** (2)**.**

**5.2 Liquid Chromatography-Mass Spectrometry Analysis**

**For the analysis of polar metabolites, samples were separated using a Vanquish ultra-high-performance liquid chromatography (UHPLC) system (Thermo Fisher Scientific, USA) equipped with a Waters ACQUITY UPLC BEH Amide column (2.1 mm × 50 mm, 1.7 μm particle size; Waters, USA). Chromatographic separation was performed using solvent A (water containing 25 mmol/L ammonium acetate and 25 mmol/L ammonia) and solvent B (acetonitrile) as the mobile phases. The column temperature was maintained at 30°C, the autosampler temperature at 4°C, with an injection volume of 2 μL and a flow rate of 0.50 mL/min. The complete LC gradient elution program was as follows: 0.00 min, 95% B; 0.25 min, 95% B; 3.50 min, 65% B; 4.00 min, 40% B; 4.50 min, 40% B; 4.55 min, 95% B; and 6.00 min, 95% B. Mass spectrometric data were acquired using an Orbitrap Exploris 120 high-resolution mass spectrometer (Thermo Fisher Scientific, USA) controlled by Xcalibur software (version 4.4). Data were collected in both full-scan MS (MS1) and data-dependent MS/MS (ddMS2) modes. The MS parameters included a sheath gas flow rate of 50 Arb, auxiliary gas flow rate of 15 Arb, capillary temperature of 320°C, MS1 resolution of 60,000, MS/MS resolution of 15,000, and stepped normalized collision energy (SNCE, 20/30/40 eV). The spray voltage was set to 3.8 kV in positive ion mode and −3.4 kV in negative ion mode. Both positive and negative electrospray ionization (ESI) modes were applied during LC–HRMS/MS acquisition to improve metabolite coverage. Untargeted LC–MS/MS analysis was performed using a data-dependent acquisition (DDA) strategy. In each MS scan cycle, the top 20 most abundant precursor ions were selected for fragmentation (ddMS2) using an intensity-dependent approach. The full MS scan range was set to m/z 70–1200.**

**5.3 Data Processing and Metabolite Identification**

**Raw mass spectrometry data acquired in vendor-specific formats were converted to mzXML format using ProteoWizard software (MSConvert). Peak extraction, feature detection, and retention time alignment were performed using an R-based data processing pipeline provided by the instrument vendor/company. Metabolite identification was conducted against DB 3.0, which integrates an in-house LC–HRMS/MS spectral library and publicly available metabolite databases, including HMDB, METLIN, and metDNA2** (3)**. The in-house spectral library was constructed based on commercially available authentic metabolite standards, while public databases were used as complementary resources for metabolite annotation. The construction workflow of the in-house LC–HRMS/MS spectral library was performed with reference to the reporting recommendations proposed by the Chemical Analysis Working Group (CAWG) of the Metabolomics Standards Initiative (MSI)** (4)**. Briefly, each reference standard was prepared individually and analyzed under identical LC–HRMS/MS platform and chromatographic conditions as biological samples. The acquired MS and MS/MS data were processed to obtain representative spectral information for each metabolite, including precursor ion information, accurate mass, MS/MS fragmentation patterns, and chromatographic retention time. During library construction, detailed chemical and experimental information for each reference standard was systematically collected and archived, including metabolite name, molecular formula, molecular weight, precursor ion information, fragment ion patterns, and retention time characteristics. The processed spectral profiles were integrated with corresponding compound metadata to generate the in-house reference spectral library, which was subsequently used for MS/MS spectral matching and metabolite annotation. The overall workflow included reference standard preparation, LC–HRMS/MS data acquisition, spectral processing, quality assessment, and metadata integration. Due to the proprietary nature of the database, the complete spectral library files and internal construction parameters are not publicly available; however, the database composition and construction strategy are provided to improve methodological transparency. Metabolite annotation was performed using DB 3.0, integrating the in-house LC–HRMS/MS spectral library with public databases (HMDB, METLIN, and metDNA2). Annotation was based on a combination of accurate mass (MS1), MS/MS spectral matching, and chromatographic retention time (RT) alignment with authentic reference standards. Detected metabolite features were first matched against the in-house spectral library. Matching criteria included precursor ion accurate mass, MS/MS fragmentation patterns, and retention time consistency. Metabolites were assigned as Metabolomics Standards Initiative (MSI) level 1 identifications only when all three parameters matched those of authentic reference standards, with acceptance criteria defined as retention time deviation within 30 s and mass error within 15 ppm. Metabolites supported by MS/MS spectral similarity but without confirmation by authentic standards were classified as MSI level 2 putative identifications. Features annotated primarily based on accurate mass matching or theoretical database comparison were assigned as MSI level 3 putative candidates. Signals lacking sufficient evidence for reliable annotation were reported as unknown compounds. Confidence levels were assigned according to MSI reporting standards and established metabolomics guidelines. After annotation, data were subjected to statistical analysis and visualization for downstream comparative metabolomic interpretation and biological functional analysis.**

**5.4 Statistical Analysis and Identification of Differential Metabolites**

**Both multivariate and univariate statistical analyses were applied to identify differential metabolites. Principal component analysis (PCA) was used to visualize overall metabolic differences among samples, while orthogonal partial least squares discriminant analysis (OPLS-DA) was employed to evaluate group separation. Variable importance in projection (VIP) scores were calculated from the OPLS-DA model. In univariate analysis, Student’s t-test or non-parametric tests were used to assess differences in metabolite abundance between groups. Metabolites were considered significantly differential when simultaneously meeting the criteria of VIP > 1, *P* < 0.05, and |Log_2_ fold change (Log_2_FC)| > 1. The differential metabolites identified between the supernatants of *H. pylori* and *S. anginosus* are listed in **Table S7**. For data visualization, heatmaps were generated using Z-score normalized metabolite abundance data. The same set of significantly differential metabolites was used for heatmap construction. Z-score normalization was applied to each metabolite prior to hierarchical clustering to ensure comparability across samples. Hierarchical clustering was performed based on Euclidean distance to visualize overall similarity patterns among samples and metabolites. Heatmap visualization was used to present the relative abundance patterns of selected metabolites across experimental groups. Statistical analyses were additionally performed using GraphPad Prism 10 software. Differences between two groups were assessed using Student’s t-test. Prior to analysis, data distribution was evaluated to determine normality. A *P*-value < 0.05 was considered statistically significant.**

**References**

1.	Wang L, Li Z, Tay CY, Marshall BJ, Gu B. 2024. Multicentre, cross-sectional surveillance of Helicobacter pylori prevalence and antibiotic resistance to clarithromycin and levofloxacin in urban China using the string test coupled with quantitative PCR. Lancet Microbe 5:e512-e513.

2.	Doppler M, Kluger B, Bueschl C, Schneider C, Krska R, Delcambre S, Hiller K, Lemmens M, Schuhmacher R. 2016. Stable Isotope-Assisted Evaluation of Different Extraction Solvents for Untargeted Metabolomics of Plants. Int J Mol Sci 17.

3.	Zhou Z, Luo M, Zhang H, Yin Y, Cai Y, Zhu ZJ. 2022. Metabolite annotation from knowns to unknowns through knowledge-guided multi-layer metabolic networking. Nat Commun 13:6656.

4.	Sumner LW, Amberg A, Barrett D, Beale MH, Beger R, Daykin CA, Fan TW, Fiehn O, Goodacre R, Griffin JL, Hankemeier T, Hardy N, Harnly J, Higashi R, Kopka J, Lane AN, Lindon JC, Marriott P, Nicholls AW, Reily MD, Thaden JJ, Viant MR. 2007. Proposed minimum reporting standards for chemical analysis Chemical Analysis Working Group (CAWG) Metabolomics Standards Initiative (MSI). Metabolomics 3:211-221.
