# Supplementary material for: Differential pro-tumorigenic effects of Helicobacter pylori and Streptococcus anginosus on AGS cells: contact-dependent versus metabolite-driven mechanisms
Source: mBio. 2026 Jul 31;17(9):e01589-26. doi: 10.1128/mbio.01589-26 (PMC13556261; doi:10.1128/mbio.01589-26)
Supplement: Supplementary file 3 [file mbio.01589-26-s0003.docx]

**Table S1** Primer and probe sequences for qPCR detection of the S. anginosus *16S rRNA* gene and human *ACTB* gene.

| Target gene | Forward primer (5’→3’) | Reverse primer  (5’→3’) | Probe  (5’→3’) |
| --- | --- | --- | --- |
| *16S rRNA* | CTAATACCGCATAACAGTATGTAACA | agatcccacgggcatactggctCTATGTATCGTCGCCTAGGTA | TTGCATCGCTAGTAGATGGACCT |
| *ACTB* | CACTCTTCCAGCCTTCCTTCC | GGATGTCCACGTCACACTTCA | CATGGAGTCCTGTGGCATCCACGAAAC |

**Table S2** qPCR cycling conditions for the detection of H. pylori in gastric fluid samples.

| Step | Cycles | Temperature (℃) | Time (min:sec) |
| --- | --- | --- | --- |
| 1 | 1 | 42 | 02:00 |
| 2 | 1 | 95 | 02:00 |
| 3 | 40 | 95 | 00:10 |
| 4 | - | 58 | 00:45 |

**Table S3** qPCR cycling conditions for the detection of H. pylori in fecal samples.

| Step | Cycles | Temperature (℃) | Time (min:sec) |
| --- | --- | --- | --- |
| 1 | 1 | 42 | 02:00 |
| 2 | 1 | 95 | 02:00 |
| 3 | 10 | 95 | 00:10 |
|  |  | 65 | 00:45 |
| 4 | 35 | 95 | 00:10 |
|  | - | 58 | 00:45 |

**Table S4** qPCR cycling conditions for the detection of *S. anginosus* in gastric fluid and fecal samples.

| Step | Cycles | Temperature (℃) | Time (min:sec) |
| --- | --- | --- | --- |
| 1 | 1 | 95 | 00:30 |
| 2 | 40 | 95 | 00:05 |
|  | - | 60 | 00:34 |

**Table S5** Primer sequences for inflammatory cytokines.

| Target gene | Forward primer (5’→3’) | Reverse primer (5’→3’) |
| --- | --- | --- |
| *IL-6* | GGCACTGGCAGAAAACAACC | GCAAGTCTCCTCATTGAATCC |
| *IL-8* | CTGGCCGTGGCTCTCTTG | CCTTGGCAAAACTGCACCTT |
| *TNF-α* | CCTCTCTCTAATCAGCCCTCTG | GAGGACCTGGGAGTAGATGAG |
| *ACTB* | CATGTACGTTGCTATCCAGGC | CTCCTTAATGTCACGCACGAT |

**Table S6** qPCR cycling conditions for inflammatory cytokine gene expression in treated cells.

| Step | Cycles | Temperature (℃) | Time (min:sec) |
| --- | --- | --- | --- |
| 1 | 1 | 95 | 00:30 |
| 2 | 40 | 95 | 00:05 |
|  |  | 55 | 00:30 |
|  |  | 72 | 00:30 |
